# Liquid plasma promotes angiogenesis through upregulation of endothelial nitric oxide synthase-induced extracellular matrix metabolism: potential applications of liquid plasma for vascular injuries

**DOI:** 10.1186/s12964-023-01412-w

**Published:** 2024-02-19

**Authors:** Sung Un Kang, Haeng Jun Kim, Sukhwal Ma, Doo-Yi Oh, Jeon Yeob Jang, Chorong Seo, Yun Sang Lee, Chul-Ho Kim

**Affiliations:** 1 Department of Otolaryngology, Department of Molecular Science and Technology, Ajou University School of Medicine, 164, World cup-ro, Yeongtong-Gu, Suwon, 443-380 Republic of Korea; 2 Medical Accelerator Research Team, Korea Institute of Radiological & Medical Sciences (KIRAMS), 75 Nowonro, Nowon-gu, Seoul, 01812 South Korea

## Abstract

**Background:**

Applications of nonthermal plasma have expanded beyond the biomedical field to include antibacterial, anti-inflammatory, wound healing, and tissue regeneration. Plasma enhances epithelial cell repair; however, the potential damage to deep tissues and vascular structures remains under investigation.

**Result:**

This study assessed whether liquid plasma (LP) increased nitric oxide (NO) production in human umbilical vein endothelial cells by modulating endothelial NO synthase (eNOS) phosphorylation and potential signaling pathways. First, we developed a liquid plasma product and confirmed the angiogenic effect of LP using the Matrigel plug assay. We found that the NO content increased in plasma-treated water. NO in plasma-treated water promoted cell migration and angiogenesis in scratch and tube formation assays via vascular endothelial growth factor mRNA expression. In addition to endothelial cell proliferation and migration, LP influenced extracellular matrix metabolism and matrix metalloproteinase activity. These effects were abolished by treatment with NG-L-monomethyl arginine, a specific inhibitor of NO synthase. Furthermore, we investigated the signaling pathways mediating the phosphorylation and activation of eNOS in LP-treated cells and the role of LKB1-adenosine monophosphate-activated protein kinase in signaling. Downregulation of adenosine monophosphate-activated protein kinase by siRNA partially inhibited LP-induced eNOS phosphorylation, angiogenesis, and migration.

**Conclusion:**

The present study suggests that LP treatment may be a novel strategy for promoting angiogenesis in vascular damage.

Video Abstract

**Graphical Abstract:**

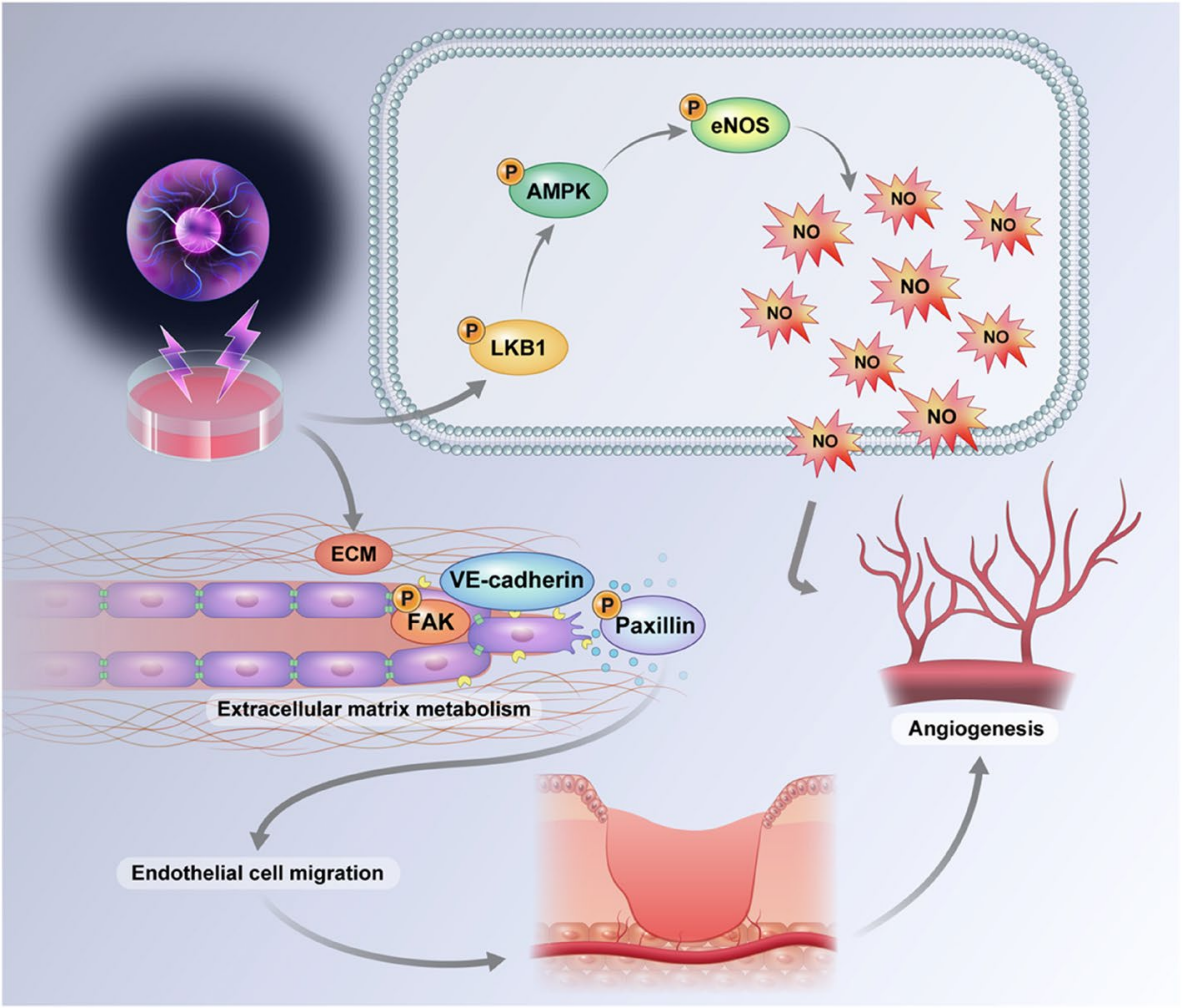

**Supplementary Information:**

The online version contains supplementary material available at 10.1186/s12964-023-01412-w.

## Background

Plasma is often referred to as the fourth state of matter [1, 2]. Technological advances have allowed the generation of liquid plasma (LP), and the treatment of living cells and tissues with LP has become possible [3, 4]. In our previous study, we demonstrated that LP is effective for muscle regeneration [5] and wound healing [6]. Further investigations examining disinfection [7] and blood coagulation [8] have been conducted. Moreover, extensive studies have revealed that non-thermal plasma alters the extracellular membrane of skin epithelial cells and promotes wound healing [5, 6, 9]. However, the use of plasma treatment for complex and deep wounds, such as those with vascular and muscle damage is limited [10].

Angiogenesis is the primary mechanism underlying wound healing. To promote angiogenesis, either the direct injection of angiogenesis-stimulating factors, such as vascular endothelial growth factor (VEGF), or the administration of related genes has been implemented [11]. However, it is difficult to enhance angiogenesis to a certain extent using these methods because the effect of gene administration is not continuous [12]; therefore, a strategy involving direct activation of the cells associated with angiogenesis has recently been attempted [13–15].

Reactive oxygen species (ROS) and reactive nitrogen species are major participants in a broad range of physiological processes, especially within the vascular system, and methods to control them have been attempted [16]. ROS include H_2_O_2_, superoxide (O2^∙-^), and hydroxyl radical, whereas reactive nitrogen species include nitric oxide (NO) and peroxynitrite (ONOO-). NO plays a major role in angiogenesis, vasodilation (a mechanism involved in regulating blood vessel tension) [17–19], and blood pressure [20, 21]. NO is also involved in endothelial cell proliferation and migration, with NO loss leading to endothelial cell dysfunction. Therefore, NO is now a target for the prevention and treatment of cardiovascular diseases in various studies examining methods to regulate its synthesis [22, 23]. NO has also emerged as an important molecule for maintaining cellular homeostasis in endothelial cells involved in wound healing [8, 24, 25]. During wound healing, NO is involved in extracellular matrix (ECM) metabolism, tissue remodeling, and angiogenesis [26–29], as evidenced by a rapid increase in NO levels during the inflammatory phase of wound healing [30].

However, despite the many clinical applications of NO, the physiological changes depend on its concentration, which is difficult to control because of its short half-life [31]. Therefore, attempts have been made to overcome these limitations via signal transduction to regulate NO production. The goal of NO regulation via signal transduction is to maximize the target site effect by preventing its rapid release [32, 33].

Endothelial nitric oxide synthase (eNOS) is the major enzyme responsible for NO production in blood vessels and regulation of vascular permeability [34, 35]. Although a complex network of pathways, numerous kinases, and many phosphorylation sites are involved in eNOS activity, the regulation and phosphorylation of Ser1177 is a critical requirement for this process [28]. Although it is generally known that physiological stress within endothelial cells stimulates the production of NO from eNOS [36], the molecular mechanisms regulating NO remain controversial. According to studies, ROS regulates the phosphorylation of eNOS, and consequently, the amount of NO present inside the cells [16, 37, 38]. In addition, eNOS activity correlates with the tyrosine phosphorylation of VE-cadherin in cultured endothelial cells [39]. This explains the mechanism underlying eNOS activity that directly affects endothelial cell migration, which is distinct from vasodilation [40, 41].

Adenosine monophosphate-activated protein kinase (AMPK), a key sensor of energy and metabolic homeostasis [42], is mainly regulated by cellular AMP, which causes a conformational change that leads to phosphorylation by upstream kinases, such as LKB1 [43]. Notably, AMPK is widely recognized for its ability to phosphorylate and activate eNOS in diverse pathophysiological contexts [44], and clinical therapeutic agents, such as statins and metformin, also modulate the activation of eNOS mediated by AMPK signaling [45, 46]. However, whether AMPK signaling is associated with endothelial cell function and cell migration remains unknown.

We hypothesized that the LP-induced upregulation of eNOS might be mediated by the FAK/Src pathway, thereby promoting angiogenesis and cell migration. To validate this hypothesis, we designed the current study in which AMPK siRNA was used to study the effects of AMPK on endothelial cell angiogenesis.

To the best of our knowledge, this is the first study to evaluate the effects of LP on endothelial cell migration and angiogenesis. Herein, we propose that LP can effectively modulate NO signaling, rendering it a useful therapeutic modality for tissue damage.

## Materials and methods

### Specification of non-thermal plasma jet system

**Fig. 1:**
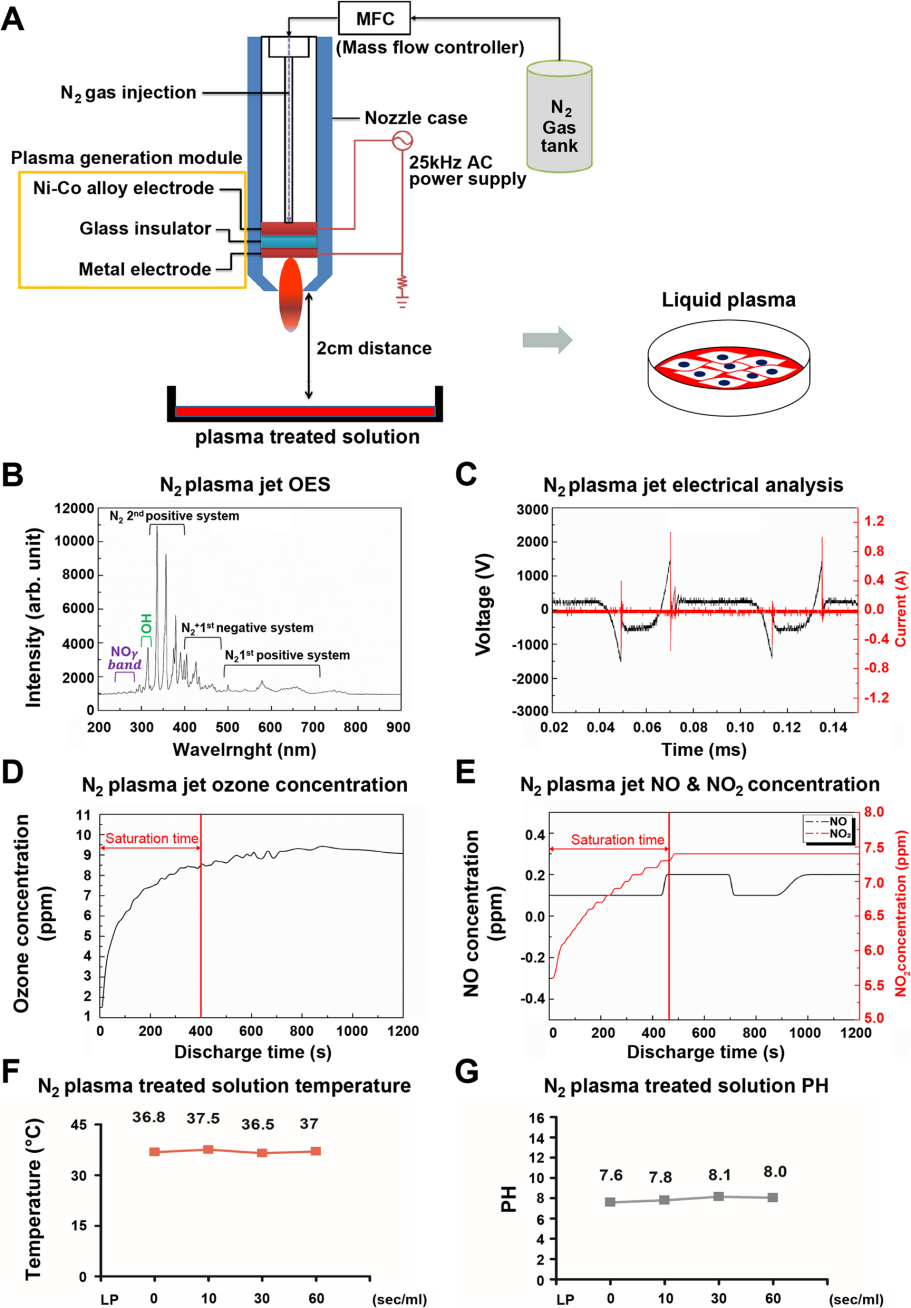
**A** Schematic drawing of the plasma apparatus and experiment configuration. **B** Optical emission spectrum in open air detected by the plasma source with 5 L/min of N_2_ gas. **C** The voltage and current waveform of the plasma jet with 5 L/min of N_2_ gas. The results show the waveform of a typical pen-type plasma jet with one current peak generated every half cycle. **D** The time-dependent concentration of ozone in open air produced by the plasma jet, as measured using an ozone analyzer. **E** The time-dependent concentration of NO and NO_2_ in the open air for the plasma jet, as measured by a gas analyzer. **F** Time-dependent effects of water temperature versus plasma treatment time. **G** Time-dependent pH values measured during the plasma treatment

We used a non-thermal plasma jet system for biomedical research. As shown in Fig. 1A, the plasma jet used in this study consisted of a nozzle case and a plasma generation module, the core components of which included Ni-Co alloy electrode, glass insulator, and electrode ring [9]. It is necessary to generate a plasma jet while maintaining a low temperature such that the surface of the biological sample is not damaged. Therefore, a nonthermal dielectric barrier discharge-type discharge method was applied. For this purpose, a dielectric material was structurally inserted between the electrodes to prevent arcing [47]. To produce plasma at atmospheric pressure, a high-voltage, low-frequency electric power module with a pulse-width modulation controller integrated circuit was employed to set the operating frequency at 25 kHz. The power supply for this system used a high-voltage transformer (1:100) to generate a 3-kV peak. A plasma jet can be generated via gas breakdown mediated by a strong electric field applied between the electrodes. Plasma temperature and density were controlled by adjusting the discharge gap, power supply, and gas flow rate. Nitrogen (N_2_) gas was used as the discharge gas at a flow rate of 5 L/min. The gas flow rate was controlled using a mass-flow controller, with a maximum mass flow rate of 10 L/min. The sample was irradiated at a distance of 2 cm from the plasma nozzle.

### Cell culture and LTP treatment

Human umbilical vein endothelial cells (HUVECs) were obtained from Lonza (CC-2935; Cell Catalog, Lonza, UK**).** Cells were maintained in endothelial growth medium (EGM-2, CC-3162, Lonza, UK) at 37 °C with 5% CO2 under humidified conditions for proliferation. HUVECs older than P6 were discarded because of the loss of their tube-formation ability. C57BL/6 Mouse Primary Vein Endothelial Cells were obtained from Cell Biologics (C57–6009, Chicago, IL, USA) and cultured according to the manufacturer’s instructions. For LTP treatment, 2 ml of the cell culture medium was added to a 6-well plate (TPP, Z707767**,** Renner, Dannstadt, Germany). The distance between the plasma device and bottom of the plate was approximately 2 cm. The LTP treatment durations were 30 and 60 sec per ml.

### Cell proliferation assay

Cell proliferation was measured with a Cell Proliferation ELISA and BrdU assay (colorimetric) (Roche Diagnostics, 11,647,229,001, Penzberg, Germany). Cell proliferation assays were performed as previously described [4]. Briefly, the HUVECs were seeded in 96-well cell culture plates at a density of 4 × 10^3^ cells/well. After 24 h, the cells were subjected to LTP treatment. Cell proliferation results are presented as ratios relative to those of untreated cells.

### Measurement of intracellular and extracellular NO

The NO levels were monitored by measuring the fluorescence changes resulting from the oxidation of 4-amino-5-methylamino-2′,7′-difluorofluorescein diacetate (DAF-FM DA; Thermo Fisher, D23844, Eu- gene, OR, USA). The cells were then incubated with the reagent according to the manufacturer’s instructions. The change in DAF-FM fluorescence was measured 24 h later using flow cytometry (BD Biosciences) and fluorescence microscopy (EVOS FL Auto, Thermo Fisher Scientific). N^G^-Methyl-L-arginine acetate salt (l-NMMA, Sigma, M7033) was used to inhibit NO generation.

Extracellular NO production was determined by measuring stable nitrite using the Griess reagent (Abcam, Cambridge, UK), according to the manufacturer’s protocol. Briefly, 50 μl of supernatant was added to a 96-well plate, followed by 50 μl sulphanilamide and 50 μl N-1-naphthyl ethylenediamine dihydrochloride. Absorbance was measured at 540 nm using a microplate reader (Epoch 2, BioTek Instruments, Inc., Winooski, VT, USA).

### Measurement of intracellular and extracellular ROS production

For the measurement of ROS production, cells were treated with LTP for 24 h and then treated with 10 μM hydroethidine (HE; Molecular Probes) as described previously [48]. Fluorescence-stained cells (1X10^4^) were analyzed using flow cytometry. To measure extracellular ROS production, the supernatant was treated with an Amplex Red assay reagent (Invitrogen, Carlsbad, CA, USA) and incubated at room temperature for 30 min. The concentration of H_2_O_2_ in the supernatant was determined using a microplate reader (Epoch 2; BioTek Instruments, Inc., Winooski, VT, USA) with excitation at 540 nm.

### Tube formation assay

HUVECs were trypsinized and then seeded into a 96-well plate (2 × 10^4^/well) precoated with 40 μl (10 mg/ml) of growth factor-reduced Matrigel (BD Biosciences, Billerica, MA) in the EBM2 medium. After 30 min, 1 h, 3 h, and 6 h of incubation at 37 °C, the cells were stained with Calcein AM (Invitrogen, Carlsbad, CA) to detect live cells and then the capillary-like structures were imaged using EVOS FL Auto (Thermo Fisher). Image densities were quantified using the National Institutes of Health ImageJ 1.41q software.

### Western blotting

Western blotting was performed as previously described [3]. Briefly, the cells were lysed with RIPA buffer (Sigma Aldrich) containing 150 mM NaCl, 1.0% Nonidet-P 40, 0.5% sodium deoxycholate, 0.1% sodium dodecyl sulfate, 50 mM Tris (pH 8.0), a protease inhibitor cocktail, and PhoSTOP (Roche Molecular Biochemicals, Basel, Switzerland). All antibodies were purchased from Cell Signaling Technology (Danvers, MA, USA), and antibodies against VE-cadherin, p-LKB1, and LKB1 were purchased from Santa Cruz Biotechnology (Santa Cruz, California, USA). Secondary antibodies (anti-rabbit IgG or anti-mouse IgG, 1:2000) were purchased from Cell Signaling Technology. Image densities were quantified using the National Institutes of Health ImageJ 1.41q software.

### Immunocytochemistry

Immunocytochemical assays were performed as previously described [1]. Briefly, cells were cultured on coverslips (Thermo Fisher Scientific, Rochester, NY, USA) and treated with LTP (60 seconds/ml) or vehicle control. The sections were covered using a coverslip and incubated with polyclonal rabbit anti-LC3B, p-FAK, and VE-cadherin (1:200; Cell Signaling Technology, Danvers, MA, USA) for 2 h and washed with PBS, followed by incubation with Alexa 488-labeled antibody (1:250; Thermo Fisher, Eugene, OR, USA) for 1 h.

After washing thrice with PBS, the slides were stained with Hoechst 33258 (Molecular Probes) and phalloidin (1:50; Molecular Probes, R415) for 15 min to counterstain the nuclei and F-actin. Coverslips were washed with PBS, mounted with Vectashield (Vector Laboratories, Inc., Burlingame, CA, USA), and analyzed using EVOS FL Auto (Thermo Fisher).

### Wound healing assay

Wound healing assays were performed as previously described [6]. Briefly, the monolayer was scratched with a sterile pipette tip (1000 μl), followed by extensive washing to remove cellular debris. The remaining cells were treated with LTP and incubated at 37 °C for 24 h. The wound on the captured image was automatically recognized and evaluated via Metamorph® NX image software (Molecular Devices, Sunnyvale, CA, USA), and the eluate of crystal violet (cat) staining was examined using light microscopy (EVOS FL Auto).

### Gelatin zymogram assay

Matrix metalloproteases were assayed using gelatin zymography as previously described [9]. Cells were cultured in a 6-well plate (Corning, Rochester, NY, USA) and treated with LTP (60 seconds/ml) or vehicle control. The supernatant (100 μl) from each sample was mixed with 1 μl of 100 mM 4-aminophenylmercuric acetate (Sigma-Aldrich), and the samples were incubated for 1 h at 37 °C. Samples were placed in sample buffer (without 2-Mercaptoethanol) for 10 min and electrophoresed on an 8% polyacrylamide gel containing 1% gelatin.

The gels were incubated in a renaturation buffer for 60 min at room temperature, followed by incubation for 18 h in 100 ml of developing buffer at 37 °C under gentle shaking. The gels were stained with Coomassie Brilliant Blue for 3 h. After decolorization in 400 ml of destaining solution (methanol, 100 ml of acetic acid, and 500 ml of distilled water), images were obtained using an image analyzer.

### Quantitative real-time PCR

Quantitative real-time PCR was performed as previously described [6]. Target genes were quantified by one-step real-time PCR using StepOnePlus™ (Applied Biosystems, Foster City, CA, USA). The qPCR primers were purchased from Qiagen (Hilden, Germany). The expression levels were normalized based on GAPDH mRNA levels.

### siRNA transfection

Transient transfection was performed using Lipofectamine 2000 reagent (Thermo Fisher Scientific) as previously described [6]. siRNA was procured from Santa Cruz Biotechnology (Dallas, TX, USA).

### Matrigel plug assay

Ten C57/BL6 male mice (Kostech Co.) were used for animal studies. HUVECs were mixed with control (without LP) or LP cells and then mixed with growth factor-reduced Matrigel (BD, USA) in a 1:1 (v/v) ratio.

The mixtures were injected subcutaneously in the right flank with 400 μl of Matrigel (BD Biosciences) in each group.

After 15 days, the mice were euthanized. The solid Matrigel plugs were carefully removed without the surrounding connective tissue and photographed. The number of endothelial cells within each plug was assessed by immunostaining with the CD31 antibody (1:500 dilution, Invitrogen, Cat #MA5–37858).

All animal experiments were performed in compliance with the guidelines of the Animal Care and Use Committee of the Korean Research Institute of Bioscience and Biotechnology (2019–0009).

### Hemoglobin assay

The hemoglobin content was analyzed by directly measuring the supernatant levels in a Matrigel plug. The optical densities of the samples were measured at 540 nm (OD540) using a microplate reader. Hemoglobin levels were determined by the Drabkin method using a commercial assay kit (Sigma-Aldrich) according to the manufacturer’s instructions.

### Statistical analysis

Data parameters were expressed as the mean ± standard deviations (SD). The statistical significance of the groups in each assay was analyzed using the Mann-Whitney U test, one-way analysis of variance, Tukey’s test, and least significant difference post hoc tests (SPSS, Chicago, IL, USA). Differences were considered statistically significant at *P* < 0.05, and statistical significance was defined as follows: * *P* < 0.05, **P < 0.01, and ***P < 0.001.

## Results

### Electrical and optical analysis of non-thermal plasma

The optical emission spectrum was used to identify various excited plasma species produced by the N_2_ plasma jet over a wide wavelength range (200–900 nm) (Fig. 1B). This emission spectrum was dominated by the presence of excited nitrogen species and could be divided into N_2_ 2nd positive, N_2_ 1st negative, and N_2_ 1st positive systems in the ranges 320–360, 370–430, and 460–690 nm, respectively [49, 50]. A strong NO γ band was detected at 200–271 nm, and the hydroxyl radical (*A*^2^Σ^+^ + *X*^2^Π) was also detected at 306–312 nm. To confirm stable plasma generation, electrical properties were analyzed using a digital phosphor oscilloscope (DPO4054B, Tektronix, Beaverton, OR, USA). The general Pen-type AC plasma jet applied in this study was driven under a frequency condition of tens of kilohertz and generated one microdischarge in the half-cycle of a sinusoidal wave [51–54]. Therefore, a single current peak was generated per half-voltage wave when the voltage decreased and current increased, as shown in Fig. 1C. The root-mean-square values of the voltage (V_*rms*_) and current (A_*rms*_) were 0.431 kV and 46.4 mA, respectively.

### Generation of active species from the plasma jet

The gas produced during plasma generation was analyzed, and as depicted in Fig. 1D and Fig. 1E, that O_3_ and NOx were generated as byproducts. The amount of ozone generated from the plasma jet was measured using an ozone monitor (106-M, 2 B Technologies, Boulder, CO, USA) with a measurement error of 0.01 ppm. A portable gas analyzer (MK9000, ECOM, Assamstadt, Germany) with a measurement error of ± 0.5 ppm was used to measure the concentration of NOx generated from the plasma jets in open air. A small amount of ozone was generated from the decomposition and recombination of oxygen in the atmosphere under the influence of a nitrogen plasma jet and generated UV radiation [55]. As shown in Fig. 1D, the initial increase in ozone concentration was due to the saturation time of the ozone analyzer. The 9 ppm concentration remained constant for approximately 20 min.

An average of 7.4 ppm of NO_2_ was generated from the plasma jet in the open air, as shown in Fig. 1E. Very little NO is generated from the plasma jet because the NO - NO_2_ conversion proceeds as follows [56]:

1 $${\textrm{O}}_2+\textrm{e}\to \textrm{O}+\textrm{O}+\textrm{e}$$

2 $$\textrm{NO}+\textrm{O}\to {\textrm{NO}}_2$$

3 $$2\textrm{NO}+{\textrm{O}}_2\to 2{\textrm{NO}}_2$$

4 $${\textrm{O}}_2+\textrm{O}\to {\textrm{O}}_3$$

5 $$\textrm{NO}+{\textrm{O}}_3\to {\textrm{NO}}_2+{\textrm{O}}_2$$

Presumably, NO was mostly converted to NO_2_ or utilized in the production of O_3_.

### Changes in the properties of N_2_ liquid plasma (LP)

The temperature changes over the course of LP device usage are shown in Fig. 1F. As measured by a type-K thermocouple (A1.T9304C, Daihan, South Korea), the starting LP-generating temperature was 36.8 °C stabilizing 10, 30, and 60 s later at 37 °C. Figure 1G depicts the change in pH over time. The pH can be changed by a chemical reaction with the active species injected into the solution and can be increased by increasing the number of OH ions generated by the decomposition of the injected ozone [57]. As confirmed by the results shown in Fig. 1G, the pH change in the solution was not significant because of the short treatment time.

### LP enhances eNOS signaling activation and capillary structure formation in HUVECs

**Fig. 2:**
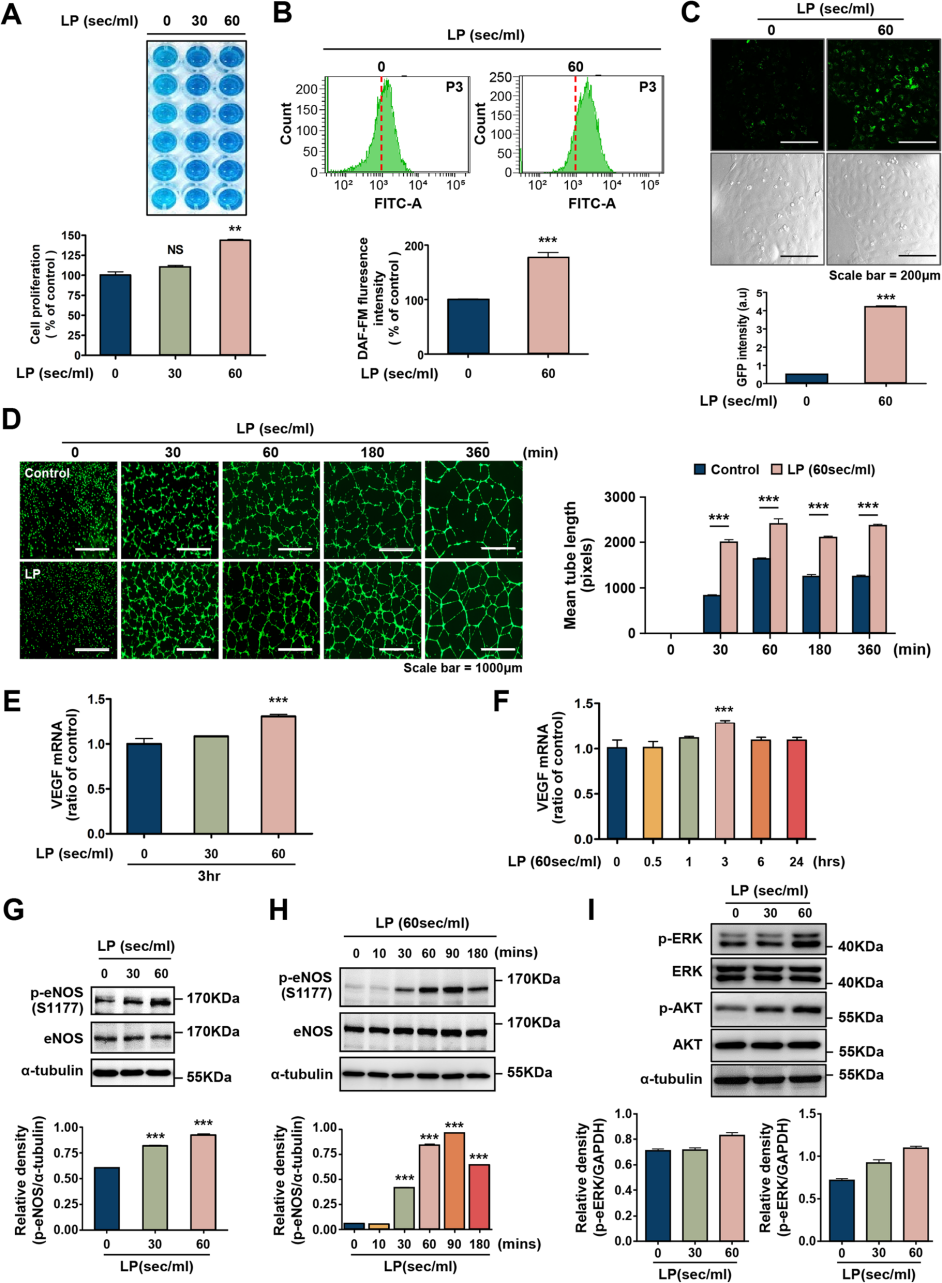
Intracellular nitric oxide detection by LP; Effects of eNOS phosphorylation and angiogenesis. **A** Cell proliferation was measured via BrdU assay. LP increased HUVEC proliferation. A statistically significant increase in the proliferation was observed with treatment time. **P < 0.01; NS = not significant. Intracellular NO analyzed by (**B**) flow cytometry, and (**C**) fluorescence images of control and LP-treated cells captured using DAF-FM probes Bar graph presents mean ± standard deviation of three independent experiments. ***P < 0.001. **D** Effects of simulated LP on tube formation in HUVEC. Representative images captured after incubation for 6 h. After cultured cells that had been incubated under control conditions or treated with LP were seeded onto Matrigel, the cells were stained with Calcein-AM and inspected under fluorescence light; scale bar = 1000 μm. Quantification of tube formation. The ImageJ plugin software was used to determine the total length of the tube-like structures in images. The bar graph shows the pixelated tube formation and the mean value. *N* = 5, ***P < 0.001. **E**, **F** The expression levels of VEGFA were measured by real-time PCR (*n* = 5, values are presented as mean ± SD). **G**, **H** eNOS signaling is involved in the LP-induced angiogenic pathway. Immunoblotting was performed to examine phosphorylated eNOS (Ser1177). LP induced the phosphorylation of eNOS (Ser1177) in a dose and time-dependent manner. Bar graph (**I**) showing the results of immunoblot analysis performed with anti-p-AKT, AKT, p-ERK, and ERK antibodies. We immediately cropped the target blots according to reference markers, and an α-tubulin antibody was used for normalization

To determine the effect of LP on endothelial cells, HUVECs were treated with LP (30 and 60 s/ml), and the effect on cell proliferation was analyzed using a BrdU assay. As shown in Fig. 2A, the degree of proliferation significantly increased in proportion to LP treatment time. The optical emission spectra of the N_2_ NTP showed that the micro-NTP jet generated excited nitrogen atoms after treatment (Fig. 1). Therefore, the intracellular NO level was measured using the fluorescent probe DAF-FM to determine whether NO generated by LP affected the activation of endothelial cells. The results showed that LP, specifically at 60 s/ml, induced intracellular NO production in HUVECs (Fig. 2B and C). Interestingly, extracellular NO levels also increased in the presence of LP (Supplementary Fig. 1A).

However, extracellular ROS levels did not increase significantly during LP treatment (Supplementary Fig. 1B). In contrast, the intracellular ROS levels significantly increased 3 h after LP treatment and decreased to the control level after 24 h (Supplementary Fig. 1C).

NO expands blood vessels, regulates cell growth, and maintains vascular homeostasis [58]. Therefore, we examined the effects of LP on proliferation and tube formation in HUVECs. We first determined the timing of the effects of LP on tube formation. LP-treated HUVECs were plated on Matrigel, and tube formation was observed at 0, 1, 3, and 6 h. A significant increase in endothelial tube formation was observed after 30 min of treatment compared to that in the controls, and the effects were more pronounced at 3 h (Fig. 2D). To determine its effect on angiogenesis, a tube formation assay was used to quantify the total tube branch and vessel thickness. The branch and vessel thicknesses of HUVEC growing on Matrigel significantly increased in the LP-treated group compared to the control group (Supplementary Fig. 4 A, B).

In addition, the increase in VEGF mRNA expression in HUVEC following plasma treatment was time-dependent (Fig. 2E), and VEGF mRNA levels peaked after 3 h of LP treatment (Fig. 2F). These results strongly suggest that LP regulates VEGF-A mRNA expression and angiogenesis.

eNOS is a key factor responsible for NO production, increased blood flow, and the regulation of angiogenic activity in vascular endothelial cells [28, 38, 59]. Therefore, to determine whether LP-generated NO is related to eNOS phosphorylation, we investigated eNOS phosphorylation at various treatment times. We first confirmed that LP induced the phosphorylation of the Ser1177 site of eNOS in a dose-dependent manner (Fig. 2G). Moreover, treatment with LP induced eNOS phosphorylation in a time-dependent manner; eNOS phosphorylation was apparent as early as 30 min after treatment and reached a maximum at 90 min (Fig. 2H).

Since eNOS can be activated by AKT/ERK [60, 61], we confirmed the phosphorylation of AKT/ERK after LP treatment. As shown in Fig. 2I, the phosphorylation of AKT and ERK increased in a dose-dependent manner. These results suggest that eNOS was phosphorylated, and intracellular NO levels increased during LP treatment. Furthermore, endothelial cell tube formation was induced.

### LP increases cell migration by regulating the extracellular matrix

**Fig. 3:**
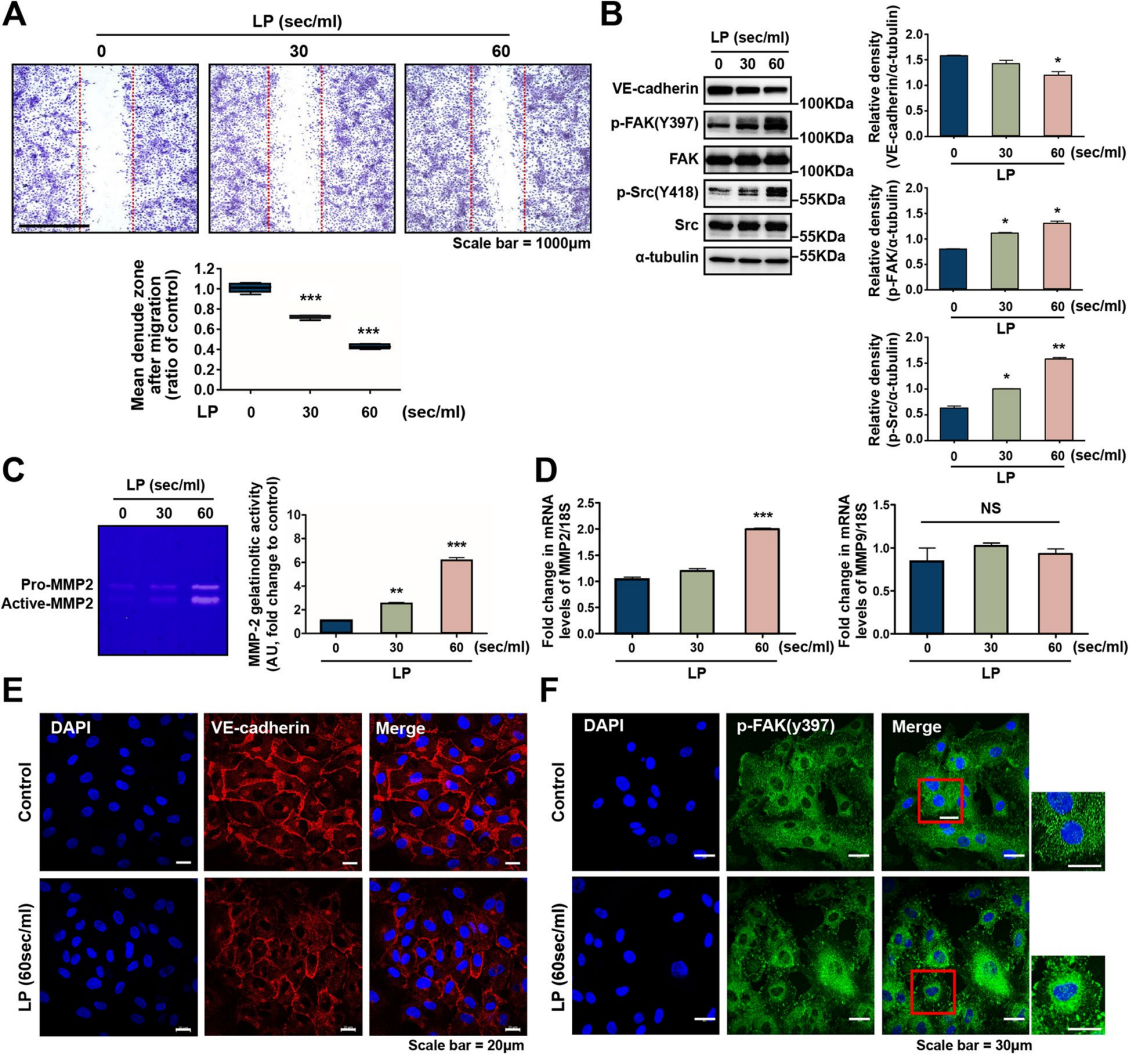
Endothelial cell migration and ECM production is enhanced by LP. **A** The effect of LP treatment on the migration potential of HUVEC was analyzed via wound migration assay. Confluent cells were wounded using a p1000 pipet tip and were either left untreated or treated with LP for 24 h. A wound migration assay showed that LP treatment for 30 s and 60 s promoted endothelial cell migration. The mean denuded zone was determined by calculating the ratio of the average denuded zone area to that of the control zone. The asterisks indicate statistically significant differences. ***P < 0.001. **B** LP regulated the total protein expression of VE-cadherin and ECM (p-FAK(Y397), FAK, p-Src(Y418), and Src) proteins, as shown by western blotting. HUVECs were treated with LP for 30 s and 60 s and then cultured for 24 h. α-tubulin was used as the loading control. Band intensities were measured and are represented as graphs. *P < 0.05, **P < 0.01. **C** A representative zymogram demonstrates that increasing concentrations of LP were associated with a selective increase in MMP-2 activity. Band quantitation performed to examine the effects of the activity of 72 kDa and 62 kDa MMP (expressed as the ratio of the control). **P < 0.01, ***P < 0.001. **D** Relative expression levels of MMP-2 and MMP-9 mRNA were determined using real-time PCR. No significant changes were observed in MMP-9 activity. **F** Immunocytochemical assays for VE-cadherin and p-FAK. **E** VE cadherin was decreased in LP-treated cells; scale bar = 20 μm. **F** Focal accumulation of FAK was significantly increased in LP-treated cells. The areas outlined in red are enlarged in the side panels; scale bar = 30 μm

Because an essential function of NO is to activate eNOS signaling pathways in endothelial cells, thereby stimulating cell growth and migration [39], we assessed whether LP had any influence on these processes. As shown in Fig. 3A, our results demonstrate that LP significantly increased the migration of HUVECs (*P* < 0.001) across the denuded zone. The percentages of migration of HUVECs were 34.2 and 58.7% after 24 h of incubation with 30 s and 60 s of LP, respectively, compared to control cells. Before wound migration analysis, the cells were pretreated with mitomycin C to inhibit cell proliferation. As shown in Supplementary Fig. 2, despite pretreatment with mitomycin C, LP-induced cell migration ability was significantly increased. These results suggest that LP affects EC migration of endothelial cells.

Furthermore, we evaluated the protein levels of VE-cadherin and phosphorylation of FAK(y397) and Src(y418), which are closely associated with cell migration, invasion, and cytoskeletal rearrangement [62, 63]. After LP treatment, we observed an increase in FAK phosphorylation, a time-dependent increase in the phosphorylation of Src, which is located downstream of FAK (Fig. 3B), and a reduction in VE-cadherin.

FAK mediates cell-matrix rearrangement by transmitting signals to MMPs, which play important roles in cell migration [9, 64, 65]. To investigate whether LP-induced mediation plays a critical role in cell migration, we performed gelatin zymography to determine MMP-2 activity. Our results revealed a noticeable increase in MMP-2 activity when HUVECs were treated with LP for 30 and 60 s compared to that in the control groups (Fig. 3C). To further identify the effect of LP on MMP-2 expression, real-time PCR was performed to examine the mRNA expression of MMP-2. As shown in Fig. 3D, LP treatment significantly increased MMP-2 mRNA expression.

Finally, the expression of VE-cadherin and p-FAK was confirmed using an immunofluorescence assay (Fig. 3E, F). VE-cadherin staining revealed reduced protein levels in the LP-treated group compared to those in the control group (Fig. 3E). In contrast, the expression of p-FAK increased in the LP-treated group but not in the control group (Fig. 3F). These findings suggest that LP increases cell migration by enhancing FAK signaling and MMP activity.

### Inhibition of eNOS attenuates LP-induced NO production, angiogenesis, and cell migration

**Fig. 4:**
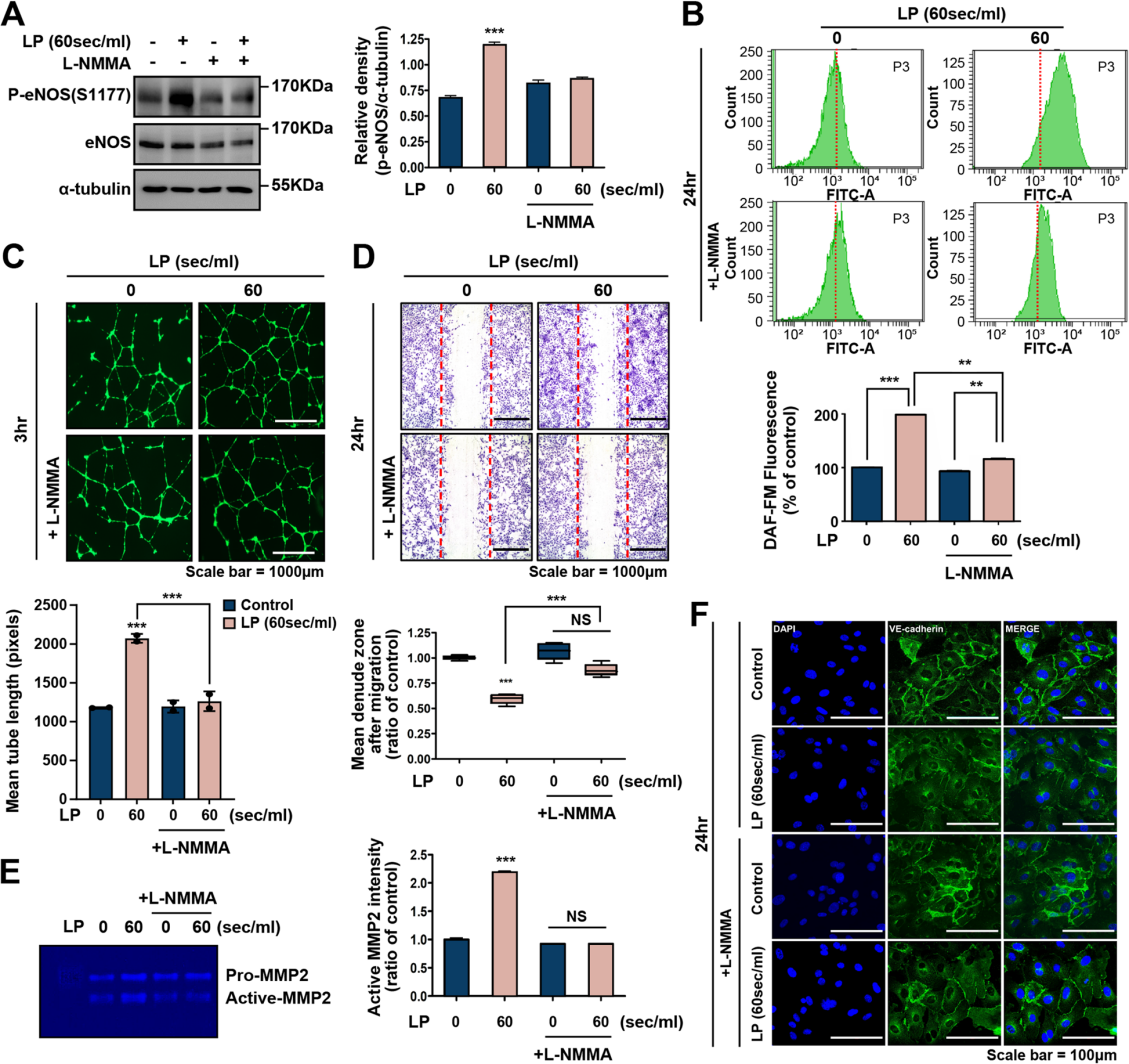
NOS inhibitors (L-NMMA, 500 μM) can selectively regulate eNOS expression, angiogenesis, and migration. **A** The expression of total eNOS and its phosphorylation were analyzed by Western blot. Phosphorylation of eNOS was upregulated after treatment with LP and was significantly attenuated by L-NMMA. The band intensities were measured and are represented as a graph. ***P < 0.001. **B** Flow cytometry using DAF-FM probes in cells treated with L- NMMA. The bar graph presents the mean ± standard deviation of three independent experiments. ***P < 0.001. **C** Angiogenic activity in LP and L-NMMA-treated HUVECs assessed using tube formation assay; scale bar = 1000 μM. Bar graph showing quantification of tube formation. *N* = 5, ***P < 0.001. Effect of LP treatment on the migratory potential of LP- and L-NMMA-treated HUVECs using (**D**) wound migration assay. Bar graph showing quantification of cell migration. ***P < 0.001, *NS* = not significant; scale bar = 1000 μM. **E** Zymogram assay. The activation of MMP-2 by LP was significantly attenuated by the L-NMMA treatment. The band intensities were measured and are represented as a graph. ***P < 0.001, *NS* = not significant, (**F**) Expression of VE-cadherin was decreased after treatment with LP and was significantly attenuated by L-NMMA; scale bar = 100 μm

To elucidate the mechanisms underlying the effects of LP on endothelial cell migration and proliferation, we evaluated the effects of LP on eNOS expression. We also analyzed LP-induced AMPK phosphorylation in HUVECs treated with the NOS inhibitor NG-monomethyl-L-arginine (L-NMMA). As shown in Fig. 4A, LP-induced eNOS phosphorylation followed by treatment with L-NMMA efficiently suppressed eNOS phosphorylation, which was also suppressed by L-NMMA (Fig 4B).

Moreover, to determine whether eNOS is involved in angiogenesis promoted by intracellular NO induced by LP, L-NMMA was added to the culture medium, and its effects were examined using a tube formation assay. As shown in Fig 4C, HUVEC tube formation was remarkably suppressed after co-incubation with L-NMMA, relative to the control group.

In addition, tube branch and vesicle thickness were remarkably suppressed after incubation with L-NMMA (Supplementary Fig. 4C, D). These results suggested that LP increased the phosphorylation of eNOS, increased intracellular NO, and enhanced tube formation in HUVECs.

Next, we explored the potency of LP in inducing eNOS phosphorylation and its association with cell migration and proliferation. eNOS regulates the migration and proliferation of endothelial cells [39]. Therefore, to investigate whether LP-induced eNOS signaling mediated cell migration and proliferation, we performed a scratch wound closure assay after L-NMMA treatment. As shown in Fig. 4D and Supplementary Fig. 3, our results demonstrate that the increased cell migration following LP treatment was reduced by L-NMMA treatment.

Moreover, the expression of MMP-2, which was markedly increased by LP treatment, was significantly reduced by L-NMMA, as shown in Fig. 4E. The migration signal of vascular endothelial cells is correlated with VE-cadherin [39]. Therefore, to further explore the relationship between NO and cell migration, VE-cadherin levels were examined using immunostaining. As shown in Fig. 4F, L-NMMA treatment efficiently increased VE-cadherin expression, which was reduced by LP treatment. These results indicate that eNOS activity and NO production by LP promote endothelial cell migration.

### AMPK stimulated by LP regulates endothelial cell migratory capacity

AMPK activation increases eNOS activity and is regulated by at least two upstream kinases, LKB1 and CaMMKKβ [59]. In particular, endothelial AMPK signaling is associated with the regulation of cell migration and proliferation under certain conditions [66].

**Fig. 5:**
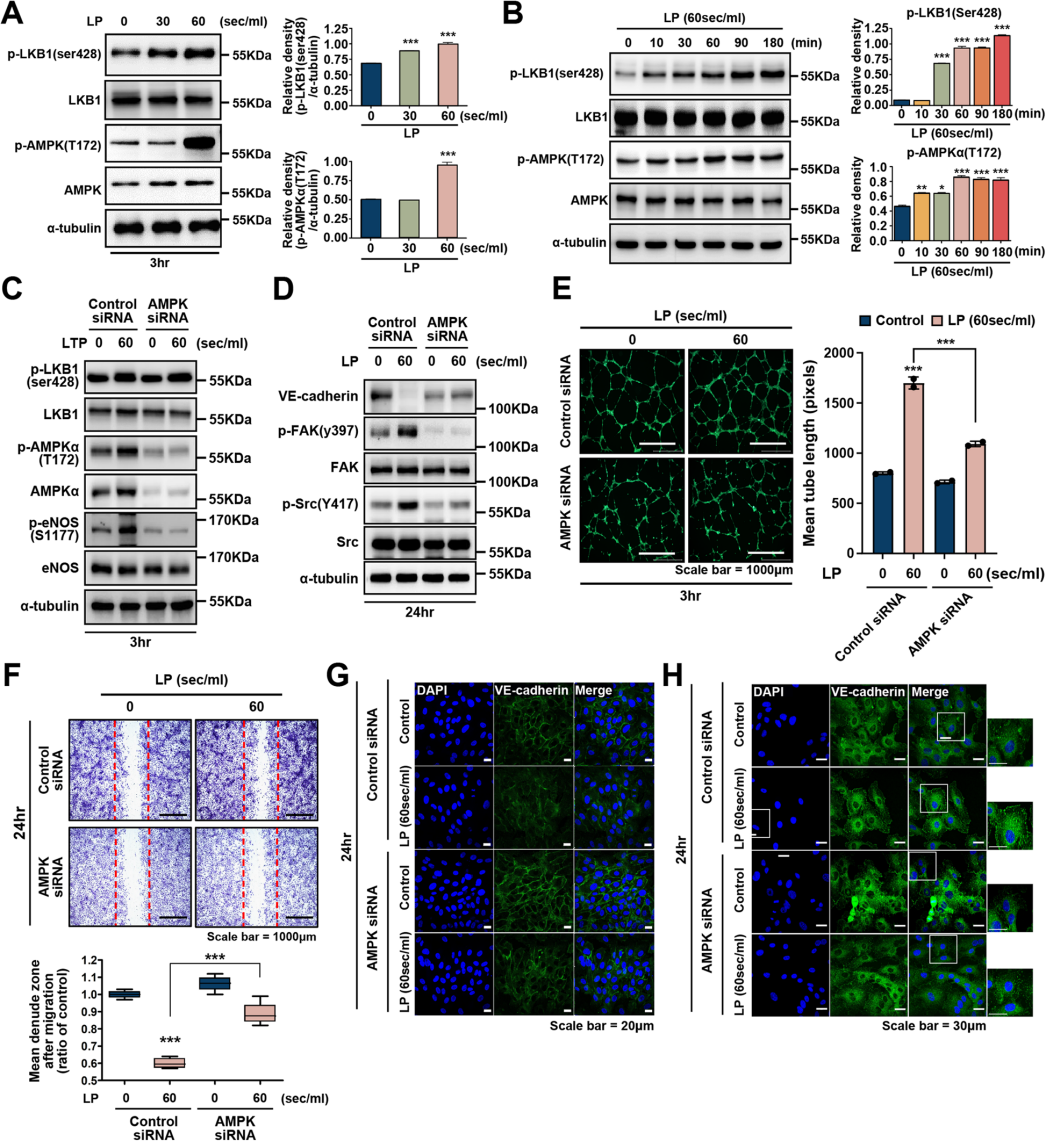
LP-induced eNOS signaling mediates phosphorylation of LKB1/AMPK. Representative western blot from a (**A**) dose-response and (**B**) time course experiment analyzed in cells stimulated with LP for 30 s and 60 s. Cell lysates were used to determine the phosphorylation of AMPK and LKB1 by western blot using antibodies specific to the phosphorylated protein. The total AMPK and LKB1 levels were also assessed as controls for loading. The band intensities were measured and are represented as a graph. ***, *P* < 0.001. LKB1/AMPK signaling regulates LP-induced angiogenesis and migration in the upstream pathway. HUVECs were transfected with AMPK-siRNA (100 pmol) or control siRNA for 24 h and then treated with LP for 60 s. **C** After 24 h, cell lysates were analyzed by western blotting using antibodies against p-AMPK, T-AMPK, p-eNOS, and T-eNOS. **D** Protein expression of VE-cadherin and ECM proteins p-FAK(Y397)), FAK, p-Src(Y418), and Src, as assessed by western blotting. **E** Angiogenic activity of AMPK siRNA-transfected HUVECs determined using a tube formation assay; scale bar = 1000 μm. Bar graph showing quantification of tube formation. *N* = 5, ***P < 0.001. **F** Migration of AMPK siRNA-transfected HUVECs in a wound migration assay. Bar graph showing quantification of cell migration. ***P < 0.001; scale bar = 1000 mM. **G**, **H** Immunocytochemical assays for VE-cadherin and p-FAK. **G** The expression of VE-cadherin was decreased upon LP treatment, and the decrease in VE-cadherin expression was mitigated in cells transfected with AMPK siRNA; scale bar = 20 μm (**H**) Expression of p-FAK was attenuated in AMPK siRNA transfected cells. The outlined areas are enlarged in the side panels. Scale bar = 30 μm

To test the role of LKB1, an upstream AMPK kinase, in AMPK phosphorylation, we measured the phosphorylation of LKB1 and AMPK in LP-treated cells. As shown in Fig. 5A, the exposure of HUVECs to LP caused a dose-dependent increase in LKB1 (Ser428) phosphorylation, with a parallel increase in the phosphorylation of Thr172 of AMPK, a well-characterized enzyme downstream of LKB, in endothelial cells [59]. Moreover, the phosphorylation of both LKB and AMPK increased upon increasing LP treatment duration from 30 min to 3 h (Fig. 5B).

Next, we used siRNA to explore the role of AMPK in LP-mediated downstream signaling. Transfection with AMPK siRNA not only significantly attenuated LP-induced p-eNOS expression (Fig. 5C) but also inhibited FAK/Src phosphorylation, which is associated with cell migration (Fig. 5D). However, the phosphorylation and total levels of the upstream signaling molecule, LKB1, were not altered. These results suggest that AMPK is required for LP-induced eNOS phosphorylation.

Next, to test whether AMPK signaling participates in LP-stimulated cell migration and angiogenesis, we used AMPK siRNA for wound migration and tube formation assays. As shown in Fig. 5E and supplementary Fig. 4E, F, AMPK siRNA significantly inhibited LP-induced endothelial tube structure formation, tube branches, and vessel thickness. Additionally, LP-stimulated endothelial cell migration was significantly suppressed by transfection with AMPK siRNA (Fig. 5F).

Finally, to confirm the association between LP-activated AMPK signaling and cell adhesion and migration, immunofluorescence microscopy was performed using antibodies against VE-cadherin and p-FAK. As shown in Fig. 5G, VE-cadherin expression was attenuated by LP but was rescued upon AMPK siRNA treatment. Moreover, the activation of p-FAK (Y397), which was increased by LP, was significantly reduced in AMPK-knockdown cells (Fig. 5H). Taken together, our results suggest that AMPK plays an important role in LP-induced endothelial cell migration and proliferation as an upstream signaling pathway.

### LP enhances vascular growth in vivo via eNOS/AMPK signaling

**Fig. 6:**
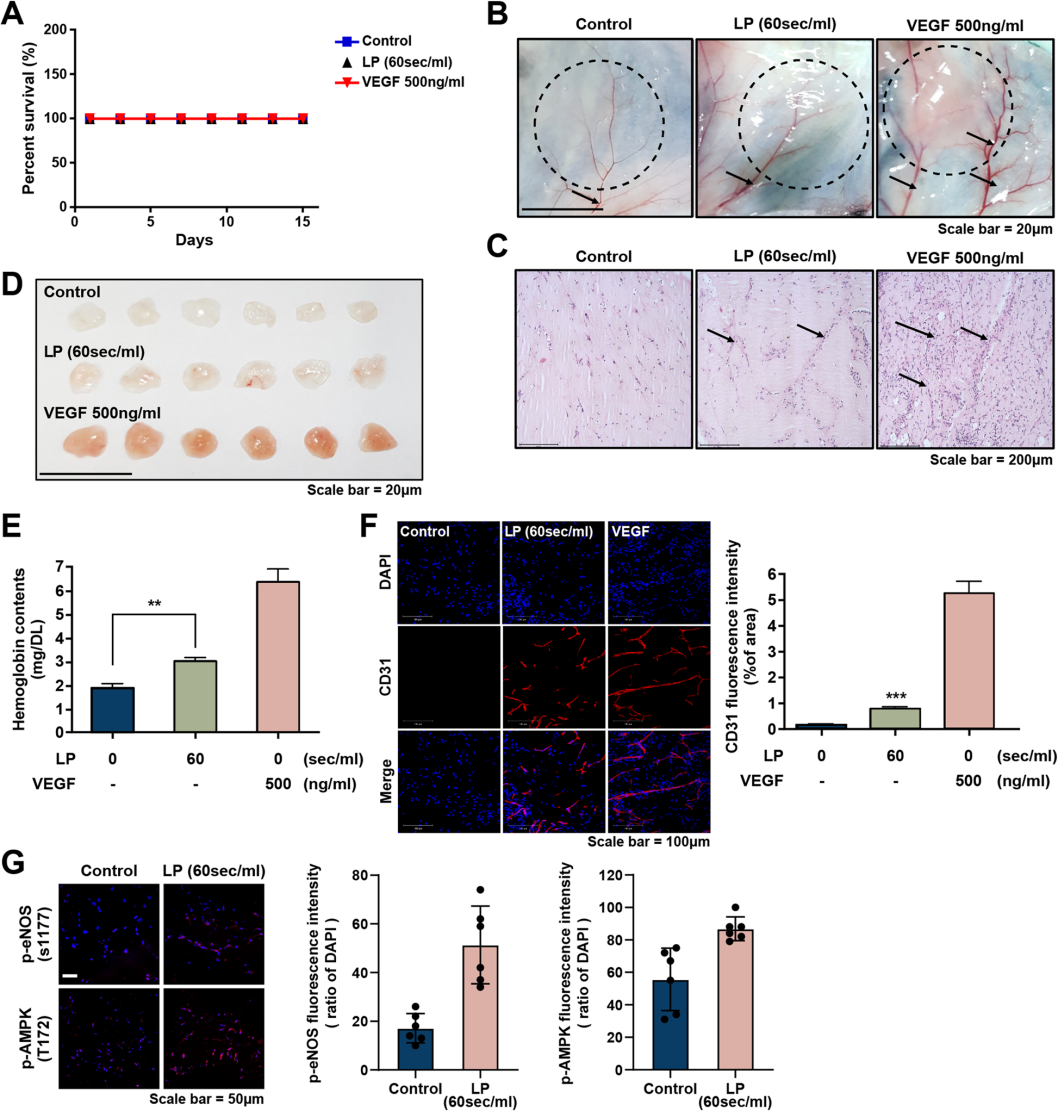
LP induces endothelial cell migration and angiogenesis in vivo. Analysis of the survival rates. No statistically significant difference was observed in the survival rate between the LP-treated group, the non-treated group (*N* = 10, each group), and the vascular endothelial growth factor (VEGF)-treated group (positive control). **B** The Matrigel plug assay. Representative photomicrographs showing angiogenesis in response to the injected Matrigel. Capillary formation towards the Matrigel was thickened in the LP-treated group compared to that in the control group; scale bar = 20 μm. (**C**) H and E staining of Matrigel plug sections from control and LP-treated mice; scale Bar = 200 μm. **D** Image shows the morphology of Matrigel plugs harvested from LP-treated and control mice; scale bar = 20 mm. **E** Hemoglobin content is shown in graphs (*N* = 10 per group). Data are presented as mean ± SEM. **P < 0.01, **P < 0.01, and ***P < 0.001 Student t test. **F**, **G** Immunofluorescence analysis of endothelial cell marker CD31 (red) and NO signaling pathway marker p-eNOS. p-AMPK levels in the sections of the Matrigel plugs derived from control and LP-treated mice are shownl nuclei are labeled using DAPI (blue); scale bar = 100 μm. Quantification of CD31-positive cells (10 fields/group), p-eNOS(S1177), p-AMPK (Thr172). ***P < 0.001

NO produced by eNOS plays an important role in vascular development and the proliferation of endothelial cells [62], but the different mechanisms by which NO regulates signaling within cells through intrinsic and exogenous pathways are unclear. Therefore, we performed a Matrigel plug assay using a control and an LP-treated cell mixture of Matrigel to determine whether LP could increase vascular recruitment. We first determined whether LP exerted cytotoxic effects. Mouse survival analysis showed that LP was not cytotoxic (Fig. 6A). As shown in Fig. 6B, the LP mixture not only increased angiogenesis but also significantly increased the thickness of the blood vessels. In addition, hematoxylin and eosin staining revealed that cells migrated around the blood vessels in the LP-treated group, unlike those in the non-treated group in vivo (Fig. 6C).

In Matrigel plugs isolated from mice, compared to those isolated from the control group, a more intense red color was observed, indicating a large number of red blood cells in the newly formed blood vessels (Fig. 6D). To quantify the extent of LP-induced angiogenesis, we measured the content of hemoglobin, an angiogenesis marker, in Matrigel. As shown in Fig. 6E, treatment with LP increased hemoglobin content by 1.6-fold. However, the combination of VEGF and LP did not increase hemoglobin content compared to a single treatment (data not shown).

Finally, microvascular density was also observed by immunocytochemical staining with the CD31 antibody, and the area of ​​CD31-positive cells was significantly increased in the LP treatment group compared to that in the control group (Fig. 6F).

To better understand the contribution of eNOS and AMPK signaling in mouse tissues, IHC analysis was performed to determine the tissue expression levels of the studied proteins.

As expected, IHC analysis revealed that a high expression of total eNOS/AMPK was noted in the cytoplasm of EC. As shown in Fig. 6G, the phosphorylation of eNOS(s1177) was significantly increased by LP. Furthermore, the expression of active AMPK (Thr172) was also significantly increased by LP treatment. These results suggested that LP induced angiogenesis via eNOS/AMPK signaling. Subsequently, we measured NO levels to assess whether the expression of NO was regulated by LP treatment via the eNOS/AMPK signaling pathway. As shown in Supplementary Fig. S6, these LP-induced signals increased tissue NO levels; however, the results were not statistically significant. Overall, our observations suggest that LP promotes angiogenesis in endothelial cells via AMPK/eNOS signaling in vivo.

## Discussion

Despite improvements in vascular wound regeneration, providing adequate wound care remains a challenge. Therefore, new therapeutic strategies are required for controlling vascular wound regeneration.

Wound healing involves vascular endothelial cells, epithelial cells, and fibroblasts, and the effects of NO on these cells have been previously studied [25, 67, 68]. Furthermore, NO enhances the migration of epithelial cells and fibroblasts both in vitro and in animals [69, 70]. However, determining the heterogeneous effects of NO on angiogenesis remains challenging. Although the angiogenic effects of NO are apparent, research on other methods is required because of the limitation of increasing NO content via ingestion [71].

A technique for the targeted inhalation-based utilization of NO is achievable through a prompt inactivation mechanism. However, only specific wounds and damaged areas can be treated, which can induce serious lung toxicity [72]. Therefore, research on activators capable of effectively generating NO is rapidly progressing, and various types of NO activators are being developed [73, 74]. In this study, we found that LP promoted vascular endothelial cell migration and regulated NO via the activation of eNOS, thereby promoting angiogenesis both in vivo and in vitro. The findings suggest that LP could be used as a novel treatment modality for several wound disorders [3, 6, 9].

Nonetheless, direct application of LTP has restricted applicability within the body and is not suitable for treatments involving large areas. Therefore, we developed a liquid form of plasma called LP [3]. We have previously revealed that LP has biological effects on wound healing and muscle differentiation [5, 9]. We also confirmed contrasting effects between normal and abnormal cells according to the type of gas used [6]. However, the underlying molecular mechanisms remain unclear. In this study, we found that LP stimulation activated the angiogenesis-signaling pathway.

Consistent with other studies, our results showed that LP upregulated eNOS activation and increased NO signaling. eNOS-derived NO plays an important role in vascular regulation in vivo and in vitro through several mechanisms, including chemical modifications [34, 39]. Several studies have confirmed the involvement of AMPK in eNOS phosphorylation. AMPK plays a vital role in the regulation of endothelial function and metabolism modulation [43, 59, 75]. AMPK activation protects endothelial cells against metabolic and inflammatory stresses [43]. Our findings confirmed that the phosphorylation of eNOS by LP was induced by AMPK activation.

Based on in vitro studies showing that LP-induced AMPK-eNOS signaling promotes the activation of vascular endothelial cells, we expected that LP would induce angiogenesis in animal models. Our in vivo Matrigel plug assay revealed that the LP-treated group showed enhanced blood vessel creation and hemoglobin content compared to the untreated group. Additionally, LP treatment significantly increased eNOS-AMPK expression and intracellular NO levels (Fig. 6G, Supplementary Fig. 5). These findings suggest that LP treatment promotes cell migration and angiogenesis, with intracellular NO levels being driven by AMPK-eNOS signaling. However, the amount of intracellular NO produced was not significantly different (Supplementary Fig. 6). These findings suggest that NO content may not persist within cells for an extended duration during in vivo experiments, and nitrite reductase activity may be, to some extent, implicated in this signaling effect [76].

In addition, we showed that the upstream kinase LKB1 was activated, which, in turn, induced eNOS phosphorylation, leading to NO production [59, 77]. Endogenous NO plays an important role in endothelial cell migration.

The present study demonstrated that plasma-induced migration and spreading of endothelial cells was accompanied by a substantial change in ECM molecules of associated signaling pathways [2]. FAK promotes cell migration via complex formation with Src and subsequent phosphorylation of the cytoskeletal adaptor molecule, paxillin, which is mediated by the FAK/Src complex [78, 79]. In a previous study, we confirmed that LP induced wound healing by mediating the expression of the FAK/Src complex in normal skin wounds [4]. Moreover, the relationship between the FAK/Src complex and the MMP-2/− 9 pathway has been previously investigated [65, 80], which showed an increased expression level of FAK/Src, consistent with the results of this study.

The effects of NTP depend on several parameters such as the gas type, size of the damage, and distance between the plasma nozzle and target tissues [9]. Nonetheless, due to its ability to permeate the epidermis, direct treatment with NTP has restricted applicability within the body [3, 81].

Hence, we created LP, a liquid plasma formulation, and ascertained that its impact mirrors that of NTP in both in vivo and in vitro settings (Fig. 1).

## Conclusions

In conclusion, we demonstrated that LP activates AMPK and NO induces migration in HUVECs. In addition, plasma-induced NO promotes the activation of ECM metabolism. Based on our results, it may be possible to develop new therapies by discovering new target molecules involved in the mechanisms of wound healing and combination therapies. Furthermore, from a clinical perspective, although the use of LP requires further investigation through in vitro and in vivo studies, our results show that LP has potential as a therapy for tissue damage.

### Supplementary Information

**Additional file 1: Supplementary Fig 1. **Effects of LP on extracellular RNS and ROS production in HUVEC cells. (A) The NO content, determined by a Griess assay kit, of HUVEC cells treated with different concentrations of LP. NO production increased in a concentration-dependent manner. Shown are mean + SD of three independent experiments.***p < 0.001. (B) H_2_O_2_ release was measured via Amplex Red assay. (C) Intracellular ROS generation was evaluated via HE staining for 30 min at 37°C. **Supplementary Fig 2.** Evaluation of cell migration by LP and the effect of mitomycin C. The Avoid cell proliferation, they were treated with 20 uM mitomycin for 1 hr before wound formation. Confluent cells were wounded using a p1000 pipet tip and were either untreated or treated with LP (60sec/ml) for 24 h. The mean denuded zone was obtained by calculating the ratio of the average area of the denuded zone to the area in the control. Asterisks indicate statistically significant differences. ***P < 0.001. **Supplementary Fig 3.** Effects of L-NMMA (NO inhibitor) on LP-stimulated HUVEC cell viability (A) and proliferation (B). Cells (1X10^4^cells/well) were plated in 96-well plates and cultured for 24 h. After 24 h, the cells were treated with LP with or without the presence of L-NMMA (500 mM). **Supplementary Fig 4.** Effect of LP on angiogenesis and angiogenesis of endothelial cells Quantification of tube formation. ImageJ plugin software was used to determine the tube branches and vessel thickness of the tube-like structures in images. (A-E) Representative bar graph is based on pixelated values of tube branches and vessel thickness. *N* = 5, *P<0.05, **P<0.01, ***P < 0.001. **Supplementary Fig 5.** Representative images of immunofluorescence staining for eNOS, AMPK (red) of matrigel sections in each group 14 days after LP treatment. Nuclei are stained with DAPI (blue) (Scale bar = 50 mm). **Supplementary Fig 6.** Levels of nitric oxide (NO) in mouse tissues. The intracellular nitric oxide content in the tissues of control and LP-treated mice was graphed. Data are presented as mean±SEM. NS=not significant. Student t test.

## Data Availability

Data supporting the findings of this study are available from the corresponding author upon request.

## Abbreviations

LP: liquid-type plasma
NO: nitric oxide
L-NMMA: NG-L-monomethyl-arginine
eNOS: endothelial nitric oxide synthase
AMPK: AMP-activated protein kinase
HUVEC: human umbilical vein endothelial cell
RNS: reactive nitrogen species
ECM: extracellular matrix
MMP: matrix metalloproteinase.
