# Supplementary material for: Liquid plasma promotes angiogenesis through upregulation of endothelial nitric oxide synthase-induced extracellular matrix metabolism: potential applications of liquid plasma for vascular injuries
Source: Cell Commun Signal. 2024 Feb 19;22:138. doi: 10.1186/s12964-023-01412-w (PMC10875778; doi:10.1186/s12964-023-01412-w)
Supplement: Supplementary file 2 — Additional file 1: Supplementary Fig 1. Effects of LP on extracellular RNS and ROS production in HUVEC cells. (A) The NO content, determined by a Griess assay kit, of HUVEC cells treated with different concentrations of LP. NO production increased in a concentration-dependent manner. Shown are mean + SD of three independent experiments.***p < 0.001. (B) H2O2 release was measured via Amplex Red assay. (C) Intracellular ROS generation was evaluated via HE staining for 30 min at 37°C. Supplementary Fig 2. Evaluation of cell migration by LP and the effect of mitomycin C. The Avoid cell proliferation, they were treated with 20 uM mitomycin for 1 hr before wound formation. Confluent cells were wounded using a p1000 pipet tip and were either untreated or treated with LP (60sec/ml) for 24 h. The mean denuded zone was obtained by calculating the ratio of the average area of the denuded zone to the area in the control. Asterisks indicate statistically significant differences. ***P < 0.001. Supplementary Fig 3. Effects of L-NMMA (NO inhibitor) on LP-stimulated HUVEC cell viability (A) and proliferation (B). Cells (1X104cells/well) were plated in 96-well plates and cultured for 24 h. After 24 h, the cells were treated with LP with or without the presence of L-NMMA (500 mM). Supplementary Fig 4. Effect of LP on angiogenesis and angiogenesis of endothelial cells Quantification of tube formation. ImageJ plugin software was used to determine the tube branches and vessel thickness of the tube-like structures in images. (A-E) Representative bar graph is based on pixelated values of tube branches and vessel thickness. N = 5, *P<0.05, **P<0.01, ***P < 0.001. Supplementary Fig 5. Representative images of immunofluorescence staining for eNOS, AMPK (red) of matrigel sections in each group 14 days after LP treatment. Nuclei are stained with DAPI (blue) (Scale bar = 50 mm). Supplementary Fig 6. Levels of nitric oxide (NO) in mouse tissues. The intracellular nitric oxide content in the tissu [file 12964_2023_1412_MOESM1_ESM.docx]

**Liquid plasma promotes angiogenesis through upregulation of e-NOS induced extracellular matrix metabolism: potential applications of liquid plasma for vascular injuries**

Sung Un Kang, Sukhwal Ma, Doo-Yi Oh, Eun Jong Ji, Jeon Yeob Jang, Chorong Seo, Yun Sang Lee, Chul-Ho Kim

**A**


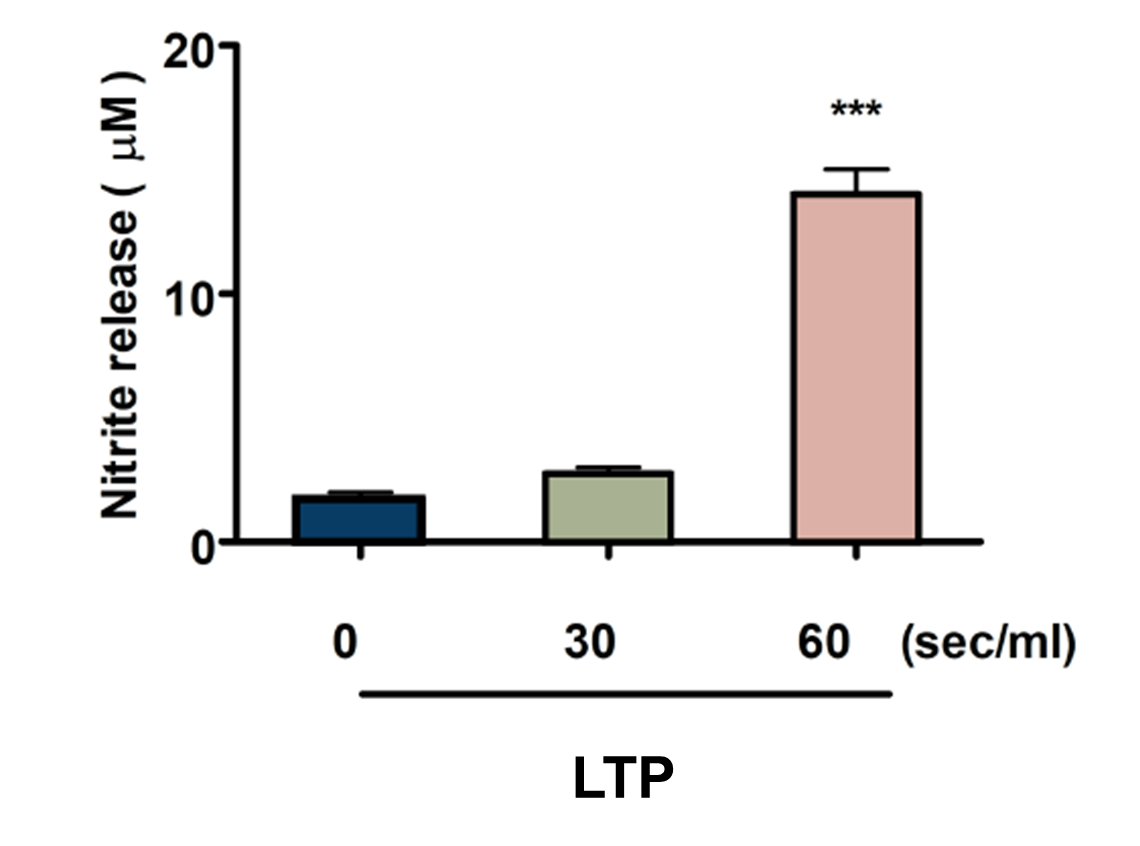

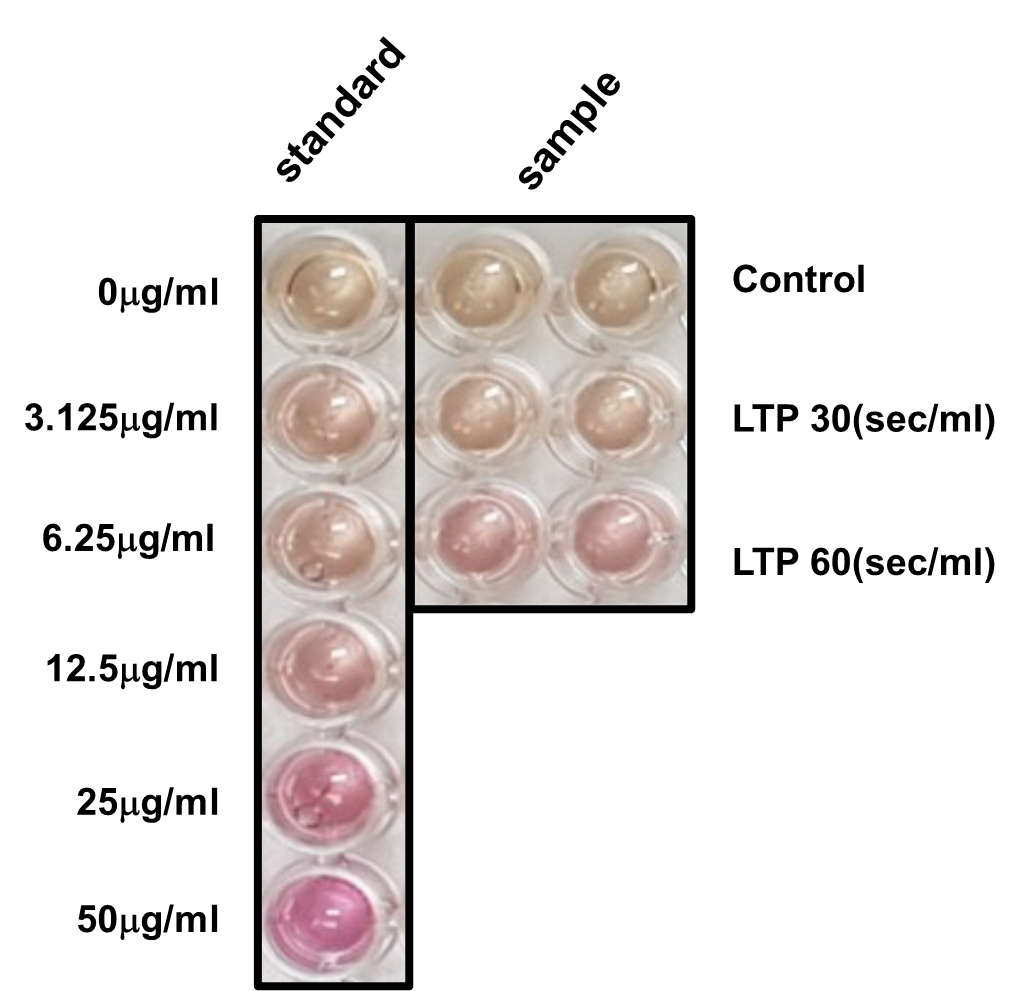


**LP**

**B**


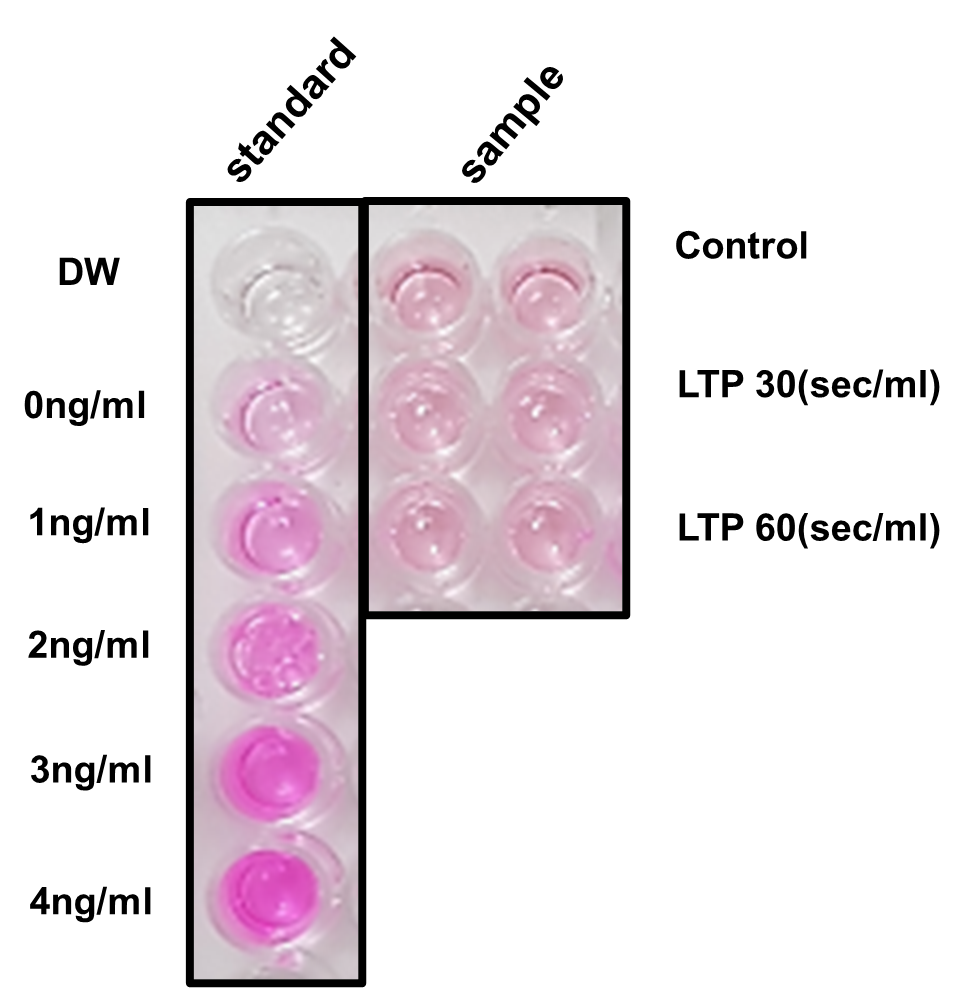

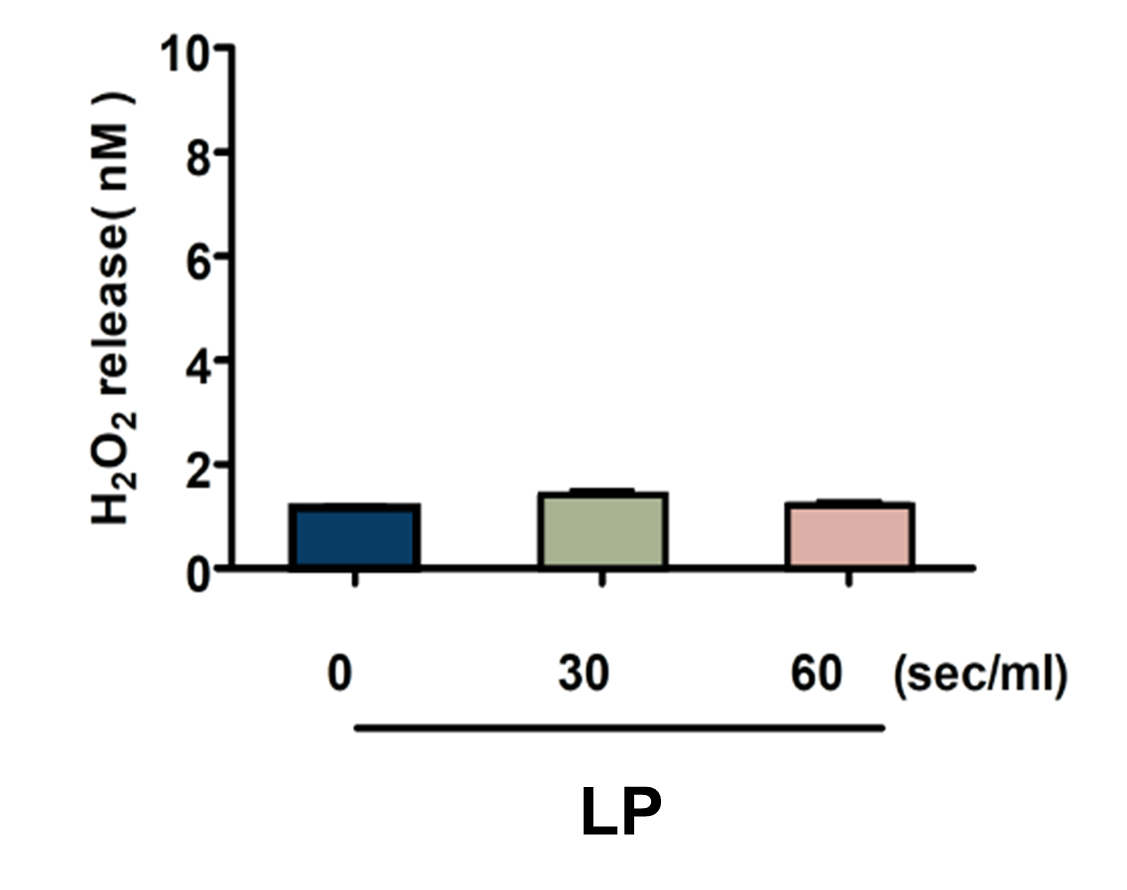


**C**


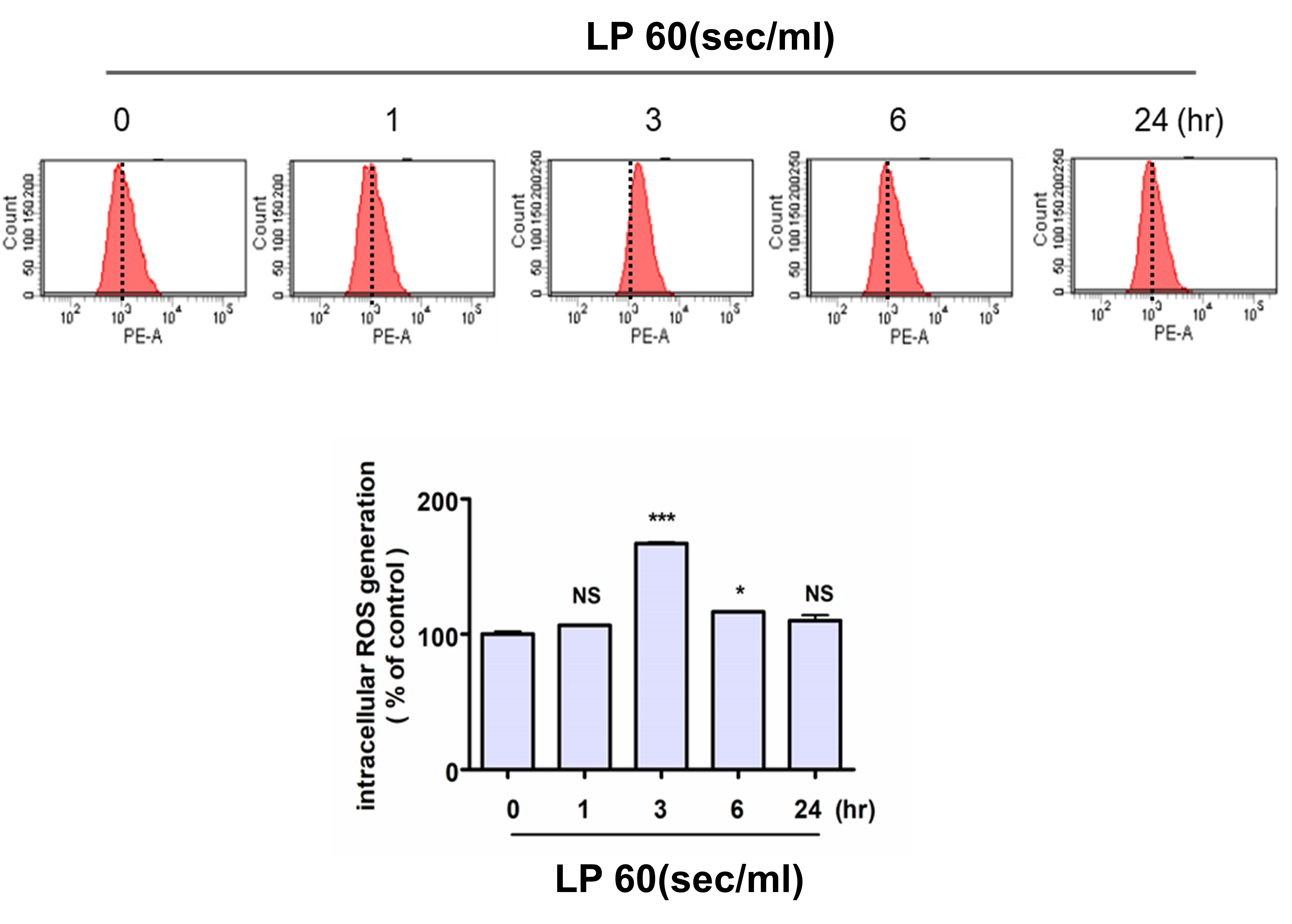


**LP 60(sec/ml)**

Supplementary Fig 1. Effects of LP on extracellular RNS and ROS production in HUVEC cells. (A) The NO content, determined by a Griess assay kit, of HUVEC cells treated with different concentrations of LP. NO production increased in a concentration-dependent manner. Shown are mean + SD of three independent experiments. ***p < 0.001. (B) H_2_O_2_ release was measured via Amplex Red assay. (C) Intracellular ROS generation was evaluated via HE staining for 30 min at 37°C.


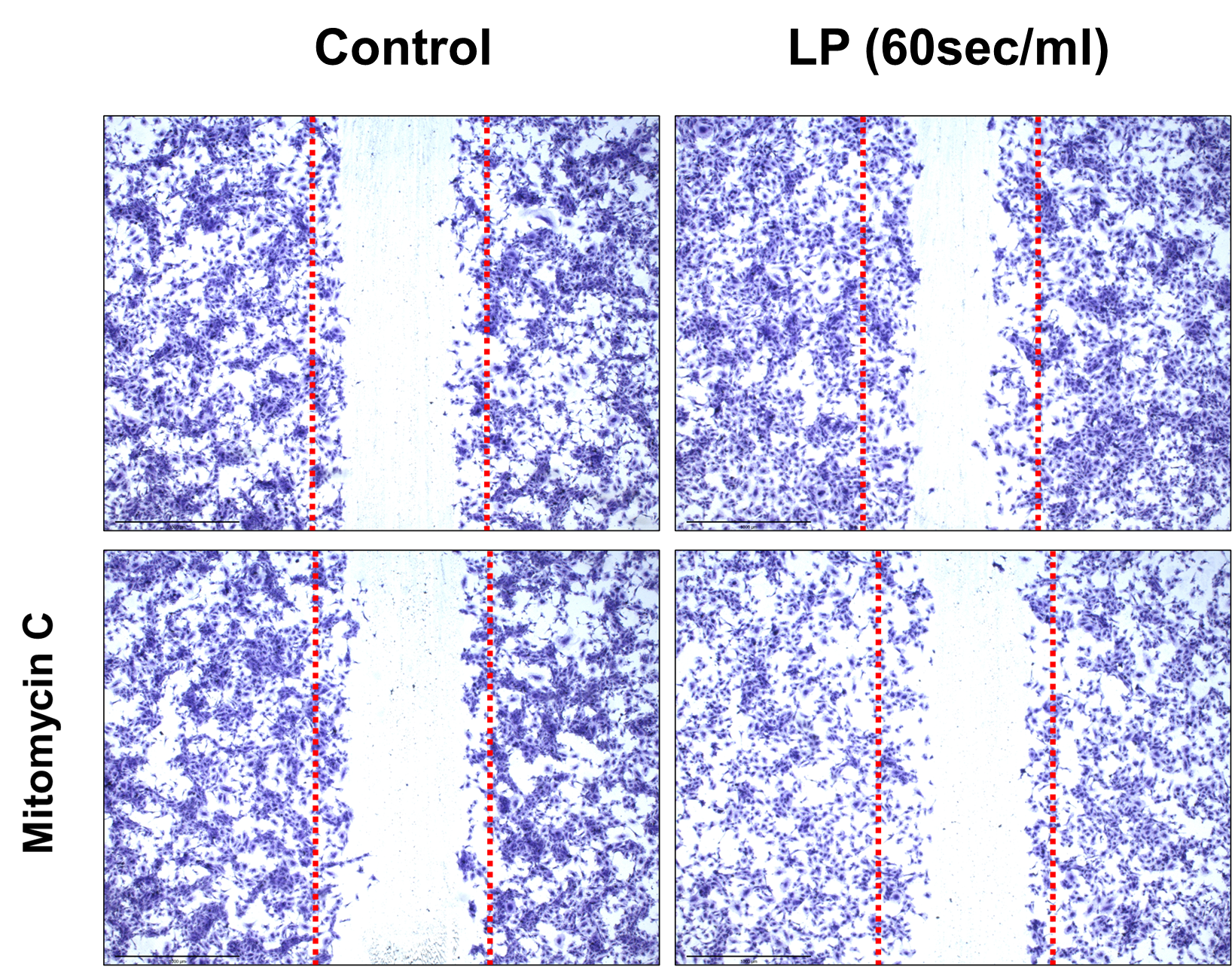


Supplementary Fig 2. Evaluation of cell migration by LP and the effect of mitomycin C. The Avoid cell proliferation, they were treated with 20 uM mitomycin for 1 hr before wound formation. Confluent cells were wounded using a p1000 pipet tip and were either untreated or treated with LP (60sec/ml) for 24 h. The mean denuded zone was obtained by calculating the ratio of the average area of the denuded zone to the area in the control. Asterisks indicate statistically significant differences. ***P < 0.001.

**A** **B**




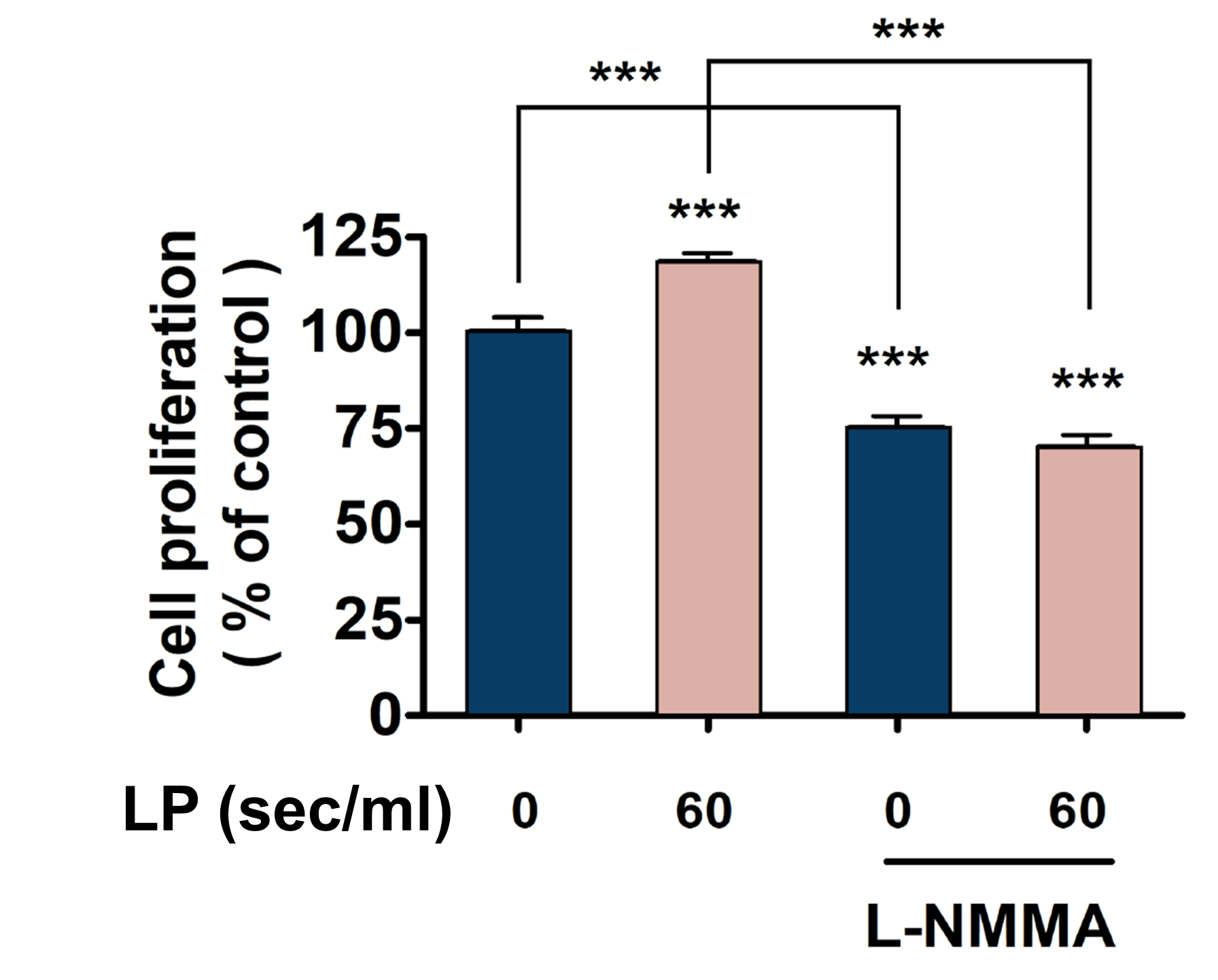


Supplementary Fig 3. Effects of L-NMMA (NO inhibitor) on LP-stimulated HUVEC cell viability (A) and proliferation (B). Cells (1X10^4^cells/well) were plated in 96-well plates and cultured for 24 h. After 24 h, the cells were treated with LP with or without the presence of L-NMMA (500 μM).


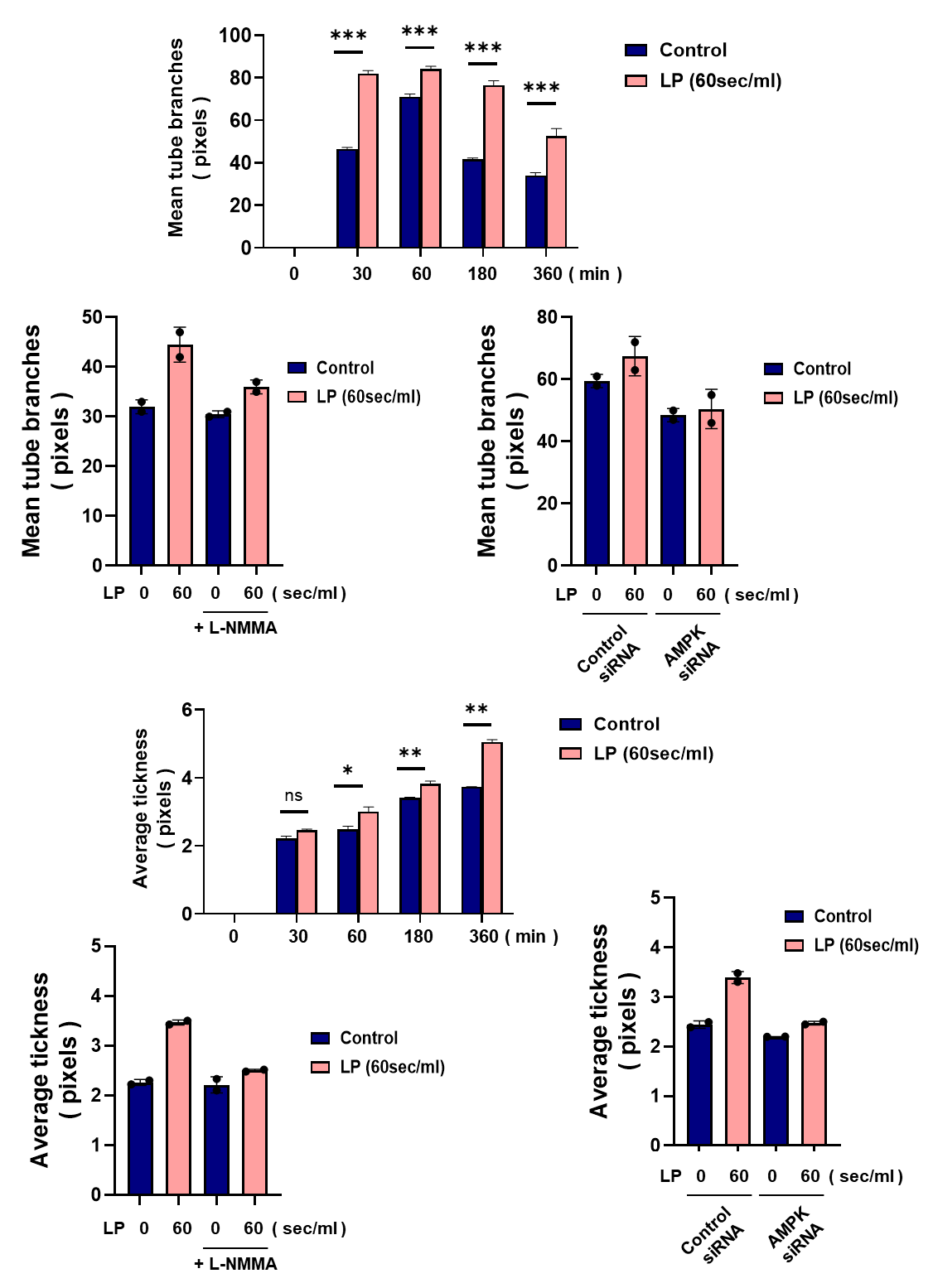


Supplementary Fig 4. Effect of LP on angiogenesis and angiogenesis of endothelial cells

Quantification of tube formation. ImageJ plugin software was used to determine the tube branches and vessel thickness of the tube-like structures in images. (A-E) Representative bar graph is based on pixelated values of tube branches and vessel thickness. N = 5, *P<0.05, **P<0.01, ***P < 0.001.


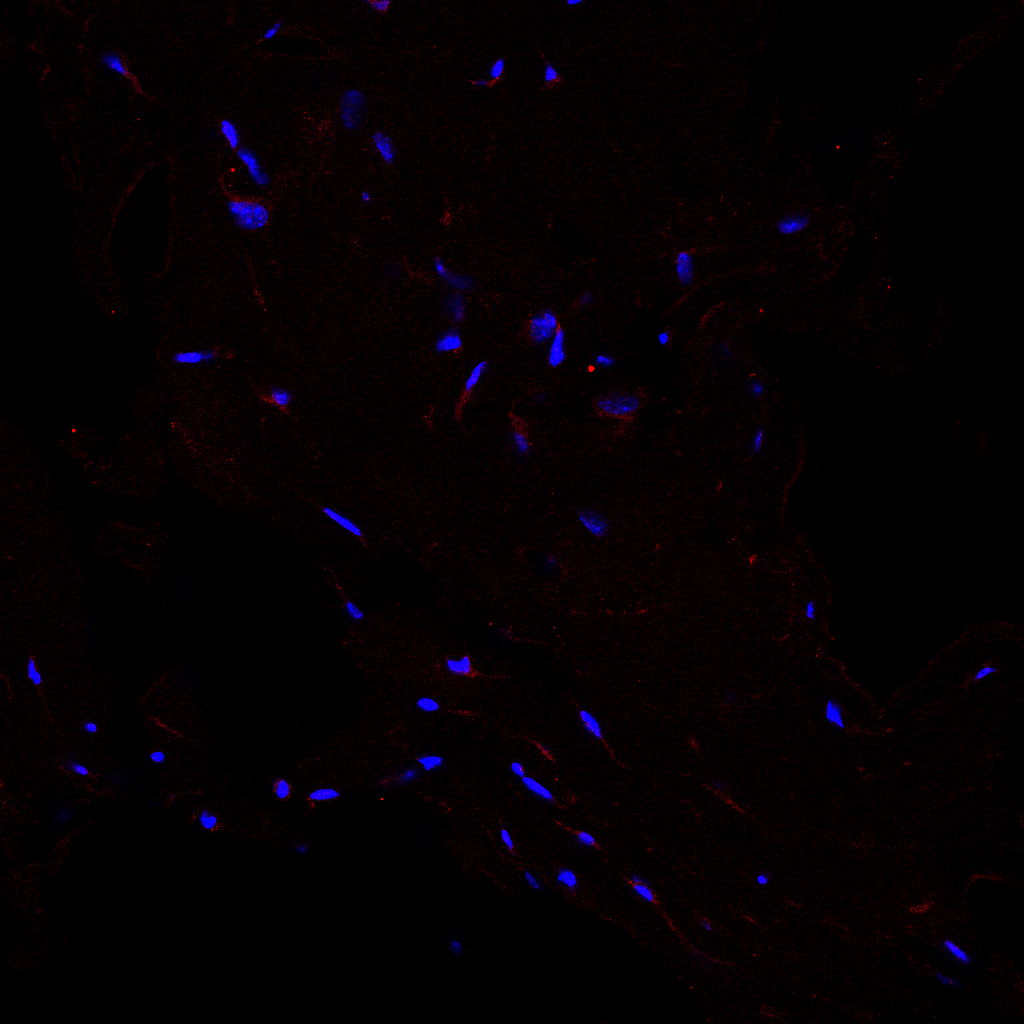

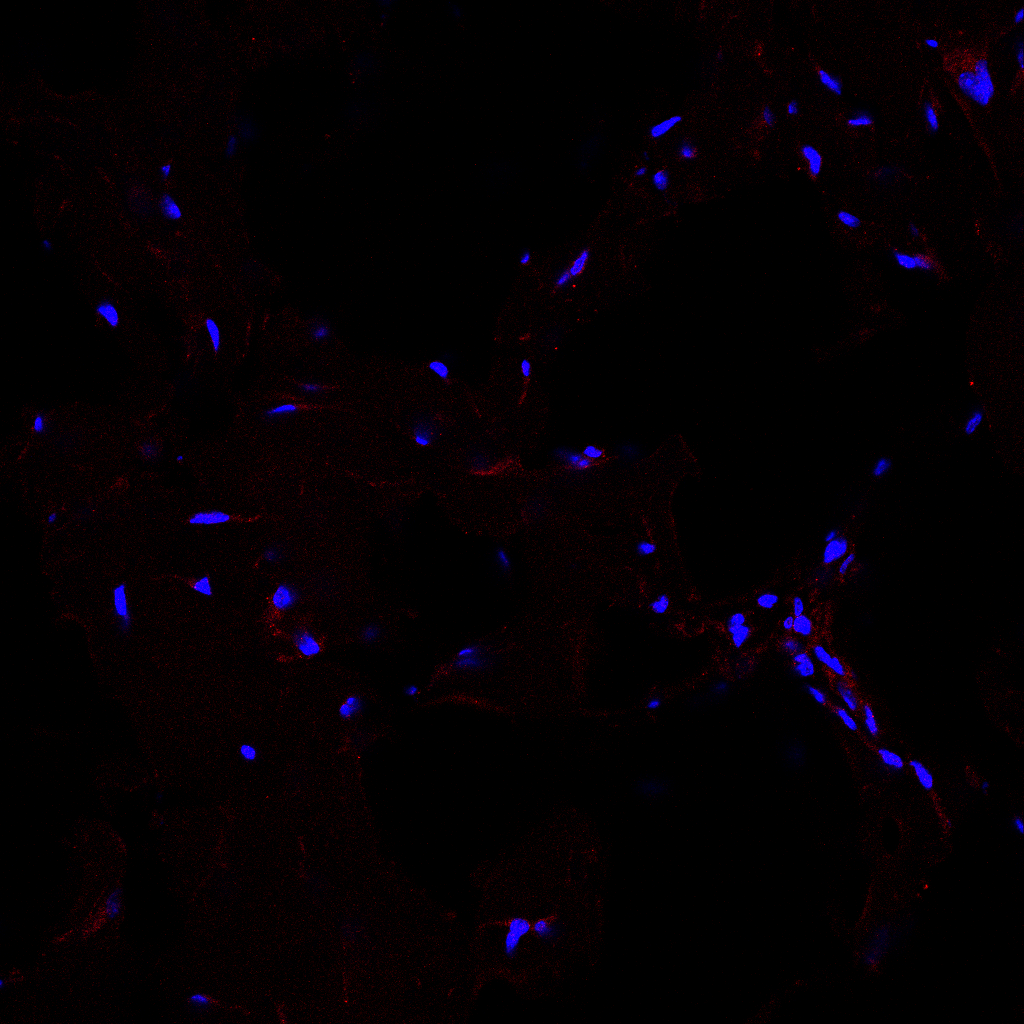

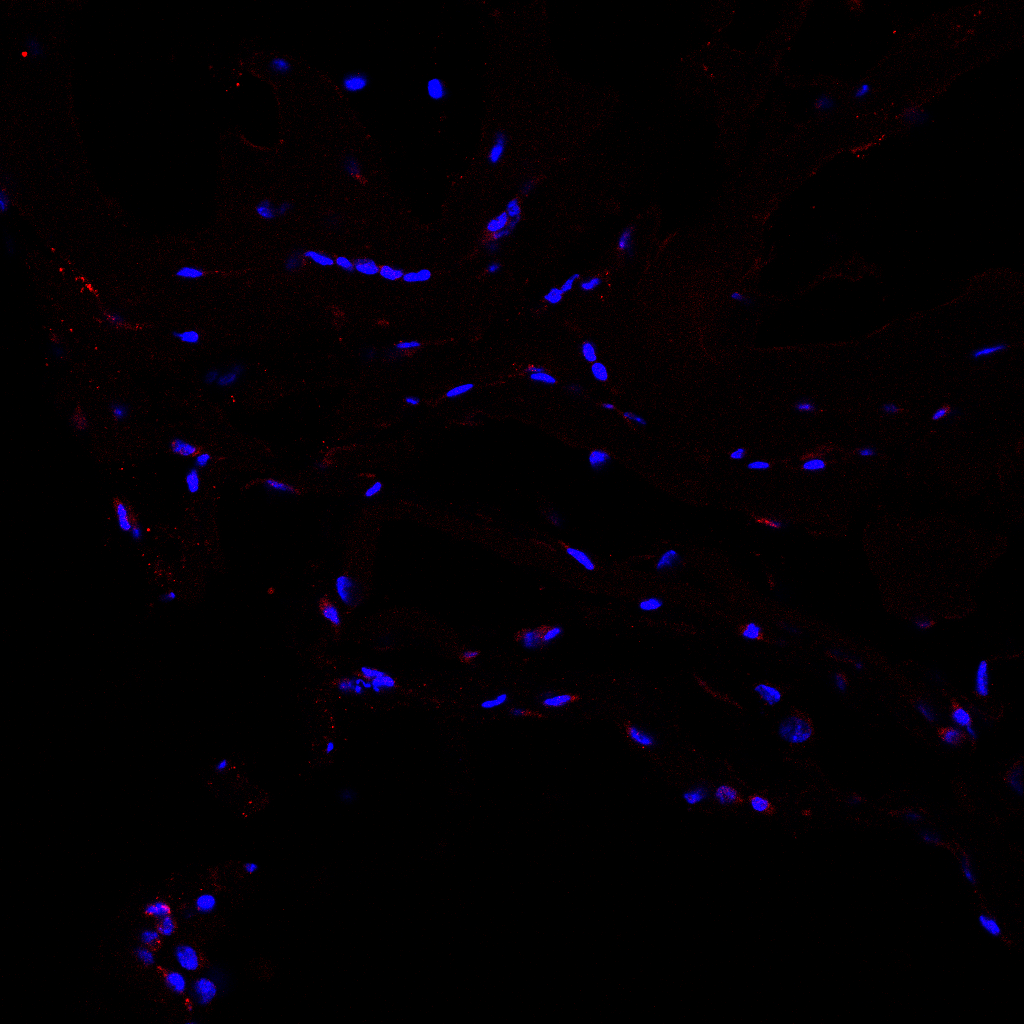

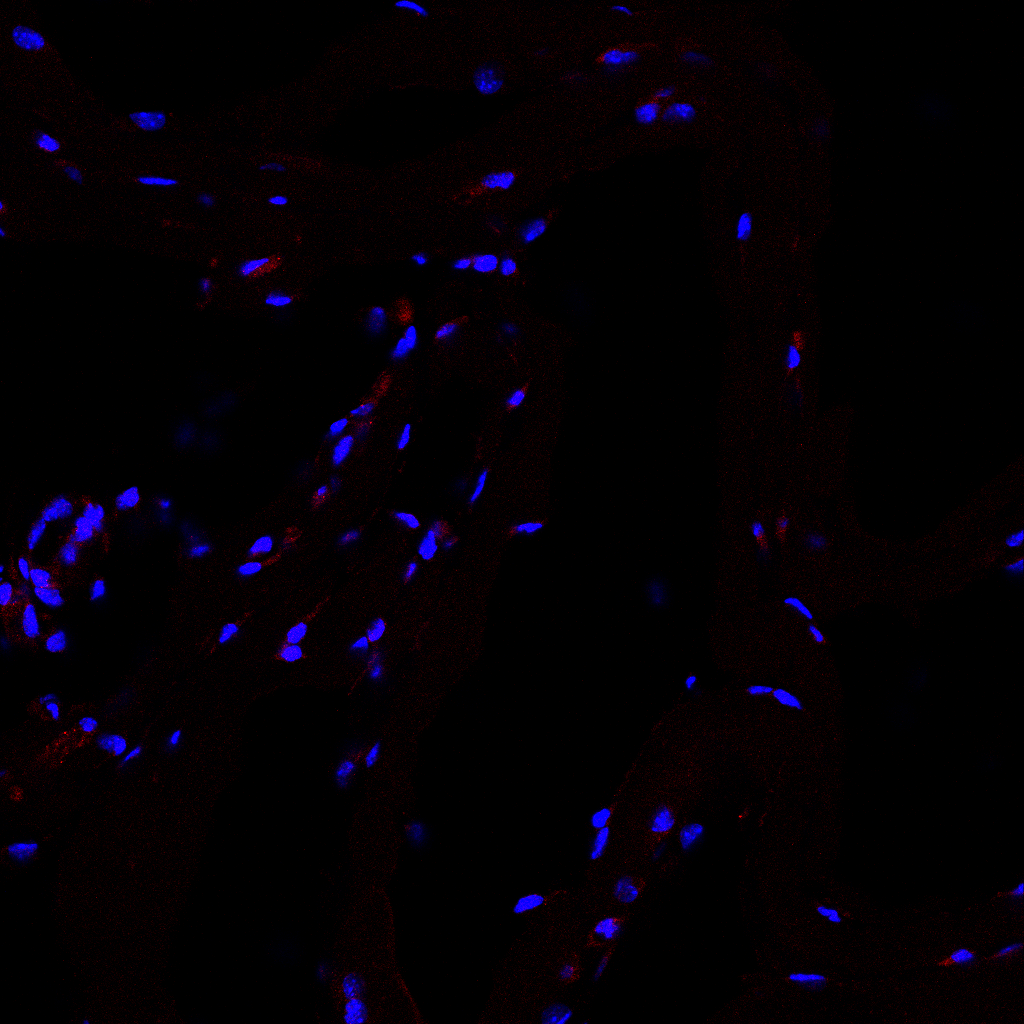


**Control**

**LP**

**eNOS**

**AMPK**

Supplementary Fig 5. Representative images of immunofluorescence staining for eNOS, AMPK (red) of matrigel sections in each group 14 days after LP treatment. Nuclei are stained with DAPI (blue) ( Scale bar = 50 μm),


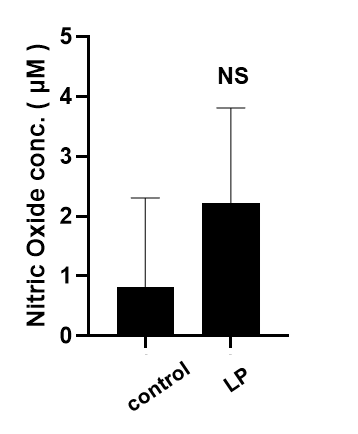


Supplementary Fig 6. Levels of nitric oxide (NO) in mouse tissues. The intracellular nitric oxide content in the tissues of control and LP-treated mice was graphed. Data are presented as mean±SEM. NS=not significant. Student t test.
